# Epigenome-wide meta-analysis of prenatal vitamin D insufficiency and cord blood DNA methylation

**DOI:** 10.1080/15592294.2024.2413815

**Published:** 2024-10-17

**Authors:** Elizabeth W. Diemer, Johanna Tuhkanen, Sara Sammallahti, Kati Heinonen, Alexander Neumann, Sonia L. Robinson, Matthew Suderman, Jianping Jin, Christian M. Page, Ruby Fore, Sheryl L. Rifas-Shiman, Emily Oken, Patrice Perron, Luigi Bouchard, Marie France Hivert, Katri Räikköne, Jari Lahti, Edwina H. Yeung, Weihua Guan, Sunni L. Mumford, Maria C. Magnus, Siri Håberg, Wenche Nystad, Christine L. Parr, Stephanie J. London, Janine F. Felix, Henning Tiemeier

**Affiliations:** a Department of Child and Adolescent Psychiatry, Erasmus MC, University Medical Center Rotterdam, Rotterdam, The Netherlands; b Generation R Study Group, Erasmus MC, University Medical Center Rotterdam, Rotterdam, The Netherlands; c Department of Psychology and Logopedics, University of Helsinki, Helsinki, Finland; d Psychology/Welfare Sciences, Faculty of Social Sciences, Tampere University, Tampere, Finland; e Lady Davis Institute for Medical Research, Jewish General Hospital, Montreal, Quebec, Canada; f Epidemiology Branch, Division of Intramural Population Health Research, Eunice Kennedy Shriver National Institute of Child Health and Human Development, Bethesda, MD, USA; g MRC Integrative Epidemiology Unit, Population Health Sciences, Bristol Medical School, University of Bristol, Bristol, UK; h Westat, Durham, NC, USA; i Centre for Fertility and Health, Norwegian Institute of Public Health, Oslo, Norway; j Section for Statistics and Data Science, Department of Mathematics, Faculty of Mathematics and Natural Science, University of Oslo, Oslo, Norway; k Department of Population Medicine, Harvard Medical School, Harvard Pilgrim Health Care Institute, Boston, MA, USA; l Faculté de médecine et des sciences de la santé, Université de Sherbrooke, Sherbrooke, QC, Canada; m Division of Biostatistics, School of Public Health, University of Minnesota, Minneapolis, MN, USA; n Department of Chronic Diseases and Ageing, Norwegian Institute of Public Health, Oslo, Norway; o Division of Intramural Research, National Institute of Environmental Health Sciences, National Institutes of Health, Department of Health and Human Services, Research Triangle Park, NC, USA; p Department of Pediatrics, Erasmus MC, University Medical Center Rotterdam, Rotterdam, The Netherlands; q Department of Social and Behavioral Sciences, Harvard T.H. Chan School of Public Health, Boston, MA, USA

**Keywords:** Vitamin D insufficiency, EWAS, PACE, DNA methylation, epigenetics, Vitamin D, pregnancy

## Abstract

Low maternal vitamin D concentrations during pregnancy have been associated with a range of offspring health outcomes. DNA methylation is one mechanism by which the maternal vitamin D status during pregnancy could impact offspring’s health in later life. We aimed to evaluate whether maternal vitamin D insufficiency during pregnancy was conditionally associated with DNA methylation in the offspring cord blood. Maternal vitamin D insufficiency (plasma 25-hydroxy vitamin D ≤ 75 nmol/L) during pregnancy and offspring cord blood DNA methylation, assessed using Illumina Infinium 450k or Illumina EPIC Beadchip, was collected for 3738 mother–child pairs in 7 cohorts as part of the Pregnancy and Childhood Epigenetics (PACE) consortium. Associations between maternal vitamin D and offspring DNA methylation, adjusted for fetal sex, maternal smoking, maternal age, maternal pre-pregnancy or early pregnancy BMI, maternal education, gestational age at measurement of 25(OH)D, parity, and cell type composition, were estimated using robust linear regression in each cohort, and a fixed-effects meta-analysis was conducted. The prevalence of vitamin D insufficiency ranged from 44.3% to 78.5% across cohorts. Across 364,678 CpG sites, none were associated with maternal vitamin D insufficiency at an epigenome-wide significant level after correcting for multiple testing using Bonferroni correction or a less conservative Benjamini–Hochberg False Discovery Rate approach (FDR, *p* > 0.05). In this epigenome-wide association study, we did not find convincing evidence of a conditional association of vitamin D insufficiency with offspring DNA methylation at any measured CpG site.

## Introduction

Vitamin D is a fat-soluble vitamin and precursor to 1,25-dihydroxyvitamin D (1,25(OH)_2_D), which plays a key role in calcium homeostasis and bone health [1]. There are two physiologically active forms of vitamin D: D_2_, found primarily in mushrooms and yeast, and D_3_, which is synthesized in the skin via UV radiation and is found in a limited number of foods, including fatty fish and egg yolks [2]. Both forms are hydroxylated in the liver to form 25-hydroxyvitamin D (25(OH)D), the major circulating form and indicator of vitamin D status, which is then hydroxylated again, primarily in the kidneys, to form 1,25(OH)_2_D [1,2].

Vitamin D concentrations are associated with a range of health outcomes, though these relationships are often nonlinear. Severely deficiencies can result in the development of rickets or osteomalacia, but extremely high concentrations (typically above 375 nmol/L) can result in vitamin D toxicity and a range of severe symptoms including recurrent vomiting, confusion, polyuria, and dehydration (though toxicity usually results from overconsumption of supplements or comorbid disorders) [3,4]. Nonlinearities have also been noted in associations between vitamin D concentrations and other health outcomes, including preterm birth and cardiovascular disease [5,6]. Individuals are typically considered to be clinically vitamin D-deficient at a 25(OH)D concentration of ≤30 nmol/L and to be vitamin D-insufficient at a 25(OH)D concentration of ≤75 nmol/L [7].

Previous research has found that vitamin D insufficiency is relatively common in pregnancy, impacting between 42.1% and 97% of pregnant study participants, depending on the location and race/ethnicity [8–10]. Maternal 25(OH)D readily crosses the placental barrier and is likely hydroxylated in fetal kidneys and the placenta itself to form 1,25(OH)_2_D [11]. Previous analyses, including in cohorts included in this study, have found associations between low maternal 25(OH)D concentrations during pregnancy and a range of offspring health outcomes, including low birthweight, small for gestational age status, bone health, attention deficit-hyperactivity disorder symptoms, autism spectrum disorder symptoms, asthma, eczema, and autoimmune conditions [12–18]. Results of randomized trials of vitamin D supplementation in pregnancy have produced mixed results, but meta-analyses have suggested that vitamin D supplementation during pregnancy may reduce the risk of low-birthweight offspring [19,20]. However, the mechanism by which maternal vitamin D levels impact offspring outcomes is not yet clear.

One possible mechanism is through offspring DNA methylation. 1,25(OH)_2_D has a known impact on gene expression through direct binding of vitamin D response elements via the vitamin D receptor transcription factor [21], but some studies have suggested that vitamin D levels may also impact DNA methylation [22,23]. To this point, research on the relationship between maternal vitamin D and offspring DNA methylation has been limited. To our knowledge, only one previous epigenome wide association study of maternal pregnancy vitamin D and offspring cord blood methylation has been conducted [24]. While the previous study did not identify any associations between maternal vitamin D and offspring methylation after adjusting for multiple tests, the study sample included 1416 mother–child pairs and was likely underpowered to detect weak or moderate epigenetic effects. The aim of this meta-analysis was therefore to investigate the associations between maternal mid-pregnancy vitamin D insufficiency and DNA methylation in the offspring cord blood in a sample expanded from the original 1416 pairs to a total of 3738 mother–child pairs.

## Methods

### Participating cohorts

This study was conducted as part of the Pregnancy and Childhood Epigenetics (PACE) consortium [25]. A total of seven cohorts participated in the study: the Avon Longitudinal Study of Parents and Children (ALSPAC); the Effects of Aspirin in Gestation and Reproduction (EAGeR) trial; the Genetics of Glucose regulation in Gestation and Growth (Gen3G) cohort; the Generation R Study; the Norwegian Mother, Father, and Child Study (MoBa1, MoBa2); the Prediction and Prevention of Preeclampsia and Intrauterine Growth Restriction (PREDO) study; and Project Viva. To limit confounding by ancestry, samples were restricted to participants of self-reported white European ancestry. We additionally excluded offspring of multiple births and offspring with known congenital abnormalities. Where multiple children of the same mother were included in a cohort, only one randomly selected child was included in the final analytic sample. Ethical approvals for all study protocols were obtained for all participating cohorts. Details on the study methods for each cohort are described in detail in the Supplementary Materials.

### Measures

#### Maternal vitamin D insufficiency

With the exception of ALSPAC, maternal vitamin D insufficiency was evaluated using serum or plasma samples taken between gestational weeks 8 and 25. Within ALSPAC, serum samples could be taken at any point during pregnancy, but all samples were normalized to obtain an estimate of concentration at 28 weeks of gestation. Maternal vitamin D sufficiency was defined as maternal serum or plasma total 25(OH)D of ≤75 nmol/L, as recommended by the Endocrine Society [7]. As noted previously, nonlinearities have been noted in associations between vitamin D concentrations and several outcomes, including preterm birth and adult cardiovascular disease [5,6]. Evidence supports an association between maternal pregnancy clinical vitamin D insufficiency at this threshold and various adverse outcomes, including preterm birth, type 1 diabetes mellitus, multiple sclerosis, allergies, and atopic disorders [26]. In order to focus our study on the impact of clinically relevant insufficiency on offspring outcomes, we chose to evaluate the relationship between dichotomous maternal vitamin D insufficiency and offspring methylation, rather than continuous 25(OH)D.

#### Offspring cord blood DNA methylation

Offspring cord blood DNA methylation was evaluated using Illumina Infinium 450k or EPIC BeadChip in all cohorts. Each cohort normalized methylation beta values using their own preferred published normalization method and conducted their own quality control pipeline for probe and sample filtering, as detailed in the Supplementary Materials. To remove outliers, methylation sets were trimmed using the interquartile range (IQR) strategy, meaning beta values below (25th percentile − 3*IQR) and above (75th percentile + 3*IQR) were removed.

#### Covariates

To improve the interpretability of findings, all models were adjusted for possible confounders of the relationship between maternal vitamin D insufficiency and offspring DNA methylation identified using subject matter knowledge [27]. All cohorts ran models adjusted for fetal sex, maternal smoking, maternal age, maternal pre-pregnancy or early pregnancy (<15 weeks gestation) BMI, maternal education, gestational age at the measurement of 25(OH)D, parity, and cell type composition. In analyses in MoBa1, MoBa2, Generation R, PREDO, and ALSPAC, maternal smoking was divided into three categories: no smoking during pregnancy, smoking during the first trimester only, and smoking throughout pregnancy. However, in EAGeR and Project Viva, insufficient data were available to apply this categorization. Within EAGeR, smoking was dichotomized into smoking during pregnancy vs. no smoking during pregnancy. Within Project Viva, the smoking status was grouped into three categories: never smoked, former smoker, and smoked during pregnancy. Maternal self-reported education was categorized according to each cohort’s discretion (see Supplementary Materials for cohort-specific details). Cell counts were estimated in each cohort using the Bakulski reference set [28]. To control ancestry, all samples except MoBa were restricted to mother–child pairs of self-reported White European ancestry. MoBa does not collect data on self-reported ancestry. However, only 5.6% of all MoBa mothers report a first language other than Norwegian, suggesting that the sample is primarily of Scandinavian ancestry [29]. Where maternal genetic data were available, models were adjusted for the first four principal components, or a number determined by the cohort to be sufficient for their sample, from DNA methylation data. In some cases, if maternal genetic data were not available, offspring principal components were used as a proxy for maternal genetic ancestry. Each cohort also adjusted for batch effects using methods appropriate to the cohort and, where necessary, included additional covariates to correct for study design (Supplementary Materials).

The primary source of vitamin D for most adults is sun exposure [1]. However, sunlight exposure, and thus vitamin D sufficiency status, varies seasonally [1]. A limited amount of research has suggested that the season of birth itself may be associated with DNA methylation [30]. As the season of 25(OH)D measurement was directly related to the season of birth, the season of measurement may confound the effect of maternal pregnancy vitamin D sufficiency on offspring methylation. In addition to stimulating vitamin D production in the skin, sunlight exposure appears to degrade folate in the skin [31–33]. Folate is a source of the one carbon group used to methylate DNA, and the maternal folate status has been associated with offspring DNA methylation in both human and animal studies [34,35]. Maternal sunlight exposure during pregnancy may therefore also impact offspring DNA methylation through folate levels. To limit this possible source of confounding, in secondary analyses, we additionally adjusted models for the season of measurement, which was grouped into four categories (February–April, May–July, August–October, and November–January), based on previous research suggesting that vitamin D sufficiency follows a seasonal pattern that lagged from astronomical seasons by approximately 8 weeks [36]. All cohorts were located in the Northern Hemisphere, meaning that they followed a similar season pattern. Because vitamin D levels in ALSPAC were pre-adjusted for season using a method described previously [37], ALSPAC results were included only in models adjusted for the season of measurement and not in the base model.

### Statistical methods

Each cohort performed independent epigenome-wide association studies according to a common pre-specified analysis plan. Associations between maternal vitamin D insufficiency and methylation at each CpG site were evaluated using two nested robust linear regression models. In the first model, offspring methylation at each CpG site was regressed on maternal mid-pregnancy vitamin D sufficiency, with adjustment for fetal sex, maternal smoking, maternal age, maternal pre-pregnancy BMI, maternal education, gestational age at measurement of 25(OH)D, parity, and cell type composition. The second model was additionally adjusted for the season of measurement.

Prior to meta-analysis, cross-reactive probes flagged by Chen et al. or McCartney et al. were removed [38,39]. We additionally removed all control and polymorphic probes as annotated by *meffil* [40] and all probes located on sex chromosomes. As methylation patterns between the Illumina 450K and EPIC arrays are highly correlated [41], analyses conducted on EPIC and 450K arrays were meta-analyzed together. Because only one cohort evaluated DNA methylation using EPIC, probes exclusive to EPIC were removed. In addition, all probes available in less than three cohorts or 1000 participants were removed. Flowcharts detailing probe removal are available in the Supplementary Materials. QQ plots, PZ plots comparing observed the p values to those calculated from the reported beta estimates and standard errors, boxplots of beta distributions, and volcano plots were generated for each analysis and visually inspected to identify possible inflation or bias of test statistics (See Supplementary Materials for results). Precision plots were generated across cohorts for each model. Fixed effects inverse-variance weighted meta-analysis of cohort specific results was conducted using *Metasoft* [19]. Correction for multiple testing was conducted using the Bonferroni method (*p* < 1.37 × 10^−7^, 364,678 tests). In addition, because the Bonferroni method can be unnecessarily conservative, we also evaluated whether associations met a significance level corresponding to the false discovery rate of 0.05 using the Benjamini–Hochberg method [42]. As a sensitivity analysis, a random effects meta-analysis of cohort specific results was also conducted using *Metasoft*. In addition to the primary meta-analysis, a shadow meta-analysis was conducted independently by authors at the University of Helsinki to minimize human error.

To characterize the top identified CpG sites, we searched the EWAS Catalog [43] to identify any previous associations between top CpG sites in our analysis and health outcomes. Using genes annotated by Illumina 450K to top CpG sites, we also conducted KEGG pathway analysis using the gometh function in the missMethyl package [44].

## Results

The prevalence of vitamin D insufficiency varied between 44.3% and 78.5% across cohorts (Table 1). In our primary analysis, with dichotomous vitamin D insufficiency as the exposure, we meta-analysed results from a total of 3239 mother–child pairs. In secondary analyses additionally adjusted for the season of measurement, we meta-analyzed results from 3738 mother–child pairs (ALSPAC participants were included only in the season of measurement models). Table 1 summarizes the characteristics of each cohort. Genomic control lambdas (base model 1.06, season of measurement model 0.93) and QQ plots (see Supplementary Materials) suggested only mild genomic inflation in the base model and did not suggest inflation in the season of measurement model.

**Table 1.** Description of included cohorts.

	ALSPAC	EAGeR	MoBa1	MoBa2	Gen3G	Generation R	PREDO	Project Viva
Country	United Kingdom	United States	Norway	Norway	Canada	Netherlands	Finland	United States
N in the largest analysis	499	361	783	177	175	1154	301	283
N (%) females	260 (52.1)	180 (49.9)	352 (45.0)	79 (44.6)	95 (54.3)	562 (48.7)	145 (48.2)	140 (49.5)
N (%) vitamin D < 75 nmol/L	306 (61.3)	162 (44.3)	389 (49.7)	91 (51.4)	128 (73.1)	706 (61.2)	198 (65.1)	222 (78.5)
N (%) any smoking during pregnancy	65 (13.0)	9 (2.5)	227 (29.0)	*Not provided by cohort*	15 (8.6)	191 (20.7)	13 (4.3)	28 (9.9)
N (%) no smoking during pregnancy	434 (87.0)	352 (97.5)	556 (71.0)	*Not provided by cohort*	160 (91.4)	731 (79.3)	288 (95.7)	255 (90.1)
Mean maternal age, years (sd) [range]	30.4 (4.4)	28.3 (4.4) [19, 40]	29.9 (4.3) [15,41]	29.4 (4.2) [18,40]	28.0 (4.1) [19-37]	31.7 (4.2) [16-46]	32.6 (5.2) [26]	32.97 (4.4) [17-44]
Mean maternal pre-pregnancy or early pregnancy BMI kg/m^2^ (sd) [range]	21.6 (7.0)	25.1 (5.3) [15.7, 50.9]	24.0 (4.2) [13.6, 43.2]	24.1 (4.7) [15.2, 43.0]	25.7 (5.8) [18.4-54.1]	23.2 (3.8) [17.3-43.3]	27.0 (6.4) [37.4]	24.9 (4.8) [16.7-50.1]
N (%) measurement Feb–Apr	107 (21.4)	86 (23.8)	115 (14.7)	36 (20.3)	55 (31.4)	277 (24.0)	59 (19.6)	68 (24.0)
N (%) Measurement May–Jul	139 (27.9)	100 (27.7)	279 (35.6)	90 (50.8)	41 (23.4)	358 (31.0)	66 (21.9)	69 (24.4)
N (%) Measurement Aug–Oct	149 (29.9)	83 (23.0)	268 (34.2)	33 (18.6)	49 (28.0)	254 (22.0)	95 (31.6)	64 (22.6)
N (%) Measurement Nov–Jan	104 (20.8)	92 (25.5)	121 (15.5)	18 (10.2)	30 (17.1)	265 (23.0)	81 (26.9)	82 (29.0)
N parity = 0	235	145 (40.2)	342 (43.7)	81 (45.8)	59 (33.7)	703 (60.9)	101 (33.6)	133 (47.0)
Gestational age of measurement	Any stage, normalized to 28 weeks	8 weeks	17-18 weeks	17-18 weeks	9.6 weeks	18-24 weeks	14-23 weeks	24-36 weeks

**Figure 1.**
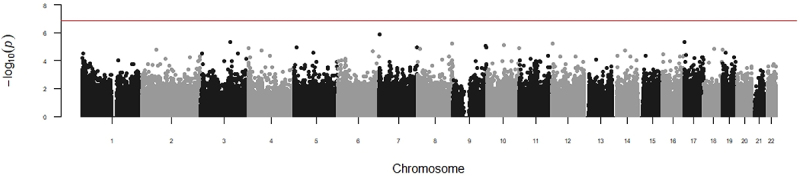
Meta-analysis of the association between maternal mid-pregnancy vitamin D and offspring DNA methylation, adjusted for fetal sex, maternal smoking, maternal age, maternal pre-pregnancy BMI, maternal education, gestational age at measurement, parity, cell type composition, principal components of ancestry, and, where appropriate, technical covariates and study design covariates. A total of 364,678 CpG sites were included in this analysis. No sites were epigenome-wide significantly associated with maternal vitamin D insufficiency using either a Bonferroni correction for multiple testing (solid line) or a Benjamini–Hochberg correction corresponding to a false discovery rate of 0.05.

**Figure 2.**
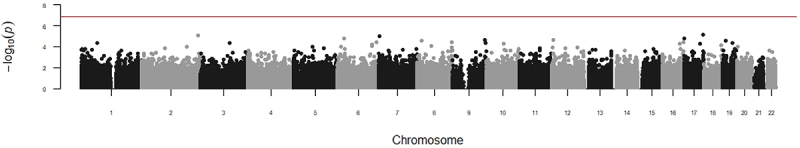
Meta-analysis of the association between maternal mid-pregnancy vitamin D and offspring DNA methylation, adjusted for the season of measurement, fetal sex, maternal smoking, maternal age, maternal pre-pregnancy BMI, maternal education, gestational age at measurement, parity, cell type composition, principal components of ancestry, and, where appropriate, technical covariates and study design covariates. No sites were epigenome-wide significantly associated with maternal vitamin D using either a Bonferroni correction for multiple testing (solid line) or a Benjamini–Hochberg correction corresponding to a false discovery rate of 0.05.

Maternal mid-pregnancy vitamin D insufficiency was not significantly associated with DNA methylation at any individual CpG site when applying a Bonferroni correction for multiple testing (*p* < 1.37 × 10^−7^) or when applying a more permissive Benjamini–Hochberg threshold (FDR <0.05) (Figure 1). P values were less than $5*10^{-5}$ at only seven CpG sites in this primary analysis. In secondary analyses, methylation was not significantly associated with maternal mid-pregnancy vitamin D sufficiency after adjustment for the season at any measured CpG site (Figure 2). Betas from both regression models tended to be small, with relatively large confidence intervals. Results of the random effects meta-analysis were broadly similar. The meta-analysis results for all probes are available in the Supplementary Materials.

Although no CpG sites were significantly associated with vitamin D insufficiency, we conducted further exploratory analyses to evaluate whether the seven CpG sites at which p values were less than 5 × 10^−5^ in our analysis were enriched in certain gene pathways or had been previously associated with other health conditions. We also conducted KEGG pathway analysis using the 500 CpG sites that met a more lenient p-value threshold of 5 × 10^−3^. KEGG pathway analysis found that no pathways were significantly differentially enriched when considering the seven CpG sites with p values less than 5 × 10^−5^ (Supplementary Table S1) or the 500 CpG sites with p values less than 5 × 10^−3^ (Supplementary Table S2). However, DNA methylation at the top seven CpG sites had been previously associated with a number of health conditions, including rheumatoid arthritis and inflammatory bowel disease (Supplementary Table S3), though these results should be interpreted with caution given the lack of significant associations with vitamin D insufficiency in our analysis.

## Discussion

In our study of mothers and children of European ancestry, we did not find evidence of a conditional association between maternal mid-pregnancy vitamin D insufficiency and offspring DNA methylation at any of the measured CpG sites after correction for multiple testing.

This study was, to our knowledge, the largest study of maternal vitamin D during pregnancy and offspring DNA methylation to date. The sample size for our primary analyses (*n* = 3239 mother–child pairs) was more than double the size of the largest previous analysis of vitamin D during pregnancy. Consistent with that previous study, this study did not find convincing evidence of an association between maternal vitamin D levels and offspring cord blood DNA methylation [24]. This lack of an association can, under the assumptions of positivity, consistency, no model misspecification, and conditional exchangeability, be interpreted as evidence that either vitamin D insufficiency does not have a causal effect on offspring DNA methylation in cord blood at any measured site or any possible causal effects of vitamin D insufficiency on offspring cord blood methylation are small [45,46]. This analysis was also less vulnerable to reverse causation than many other EWAS designs because maternal vitamin D sufficiency status during pregnancy occurs prior to offspring DNA methylation in cord blood (measured at birth), and fetal DNA methylation is relatively unlikely to affect the maternal vitamin D status during pregnancy.

However, we were only able to estimate associations for 364,678 of the approximately 28 million CpG sites contained within the human genome. Although the Illumina 450k array is targeted at regions with potential regulatory functions, it is possible that maternal vitamin D insufficiency impacts offspring DNA methylation at other genetic regions. In particular, previous studies have argued that distal regulatory elements, including enhancers, are severely underrepresented on the 450K [47,48]. While previous work has suggested that EWAS designs including more than 1000 participants are well-powered to detect moderate and small effects [49], it is also likely that our sample size, while largely relative to previous studies of maternal vitamin D and offspring DNA methylation, remains too small to detect very weak effects on offspring methylation. This issue may have been exacerbated by the use of planned meta-analysis rather than a mega-analysis pooling all raw data together, which was necessary to adhere to data-sharing regulations governing the included cohorts. Within the cohorts included in this analysis, the prevalence of vitamin D insufficiency ranged from 44.3% to 78.5%. This suggests that our analysis was not limited by a low prevalence of the exposure but rather that any possible associations of vitamin D insufficiency with methylation are small and would require larger sample sizes for detection. It is also possible that vitamin D deficiency (25(OH)D < 30 nmol/L), a more extreme exposure, may be more strongly associated with offspring DNA methylation than vitamin D insufficiency. Within our study, maternal vitamin D deficiency affected 0.0–8.1% of participants in each cohort, meaning it was too rare to conduct meaningful analyses (Supplementary Table 3), but future research in other populations could evaluate how the deficiency status in pregnancy affects offspring DNA methylation.

Other limitations of our study include possible selection bias. The majority of cohorts in this meta-analysis had a modest participation rate and measured methylation only within a subset of their total sample. If vitamin D insufficiency or offspring DNA methylation were differentially associated with selection into the sample, this could have resulted in bias [50]. Previous studies have found inconsistent associations between markers of socioeconomic status and vitamin D insufficiency, though these associations may be partially explained by differences in the racial/ethnic background of participants in different socioeconomic groups [51–54]. Because some of the studies included in this analysis show evidence of selection on socioeconomic status, a true association between vitamin D insufficiency and socioeconomic status could have resulted in selection bias [55,56]. Even if DNA methylation or vitamin D insufficiency were unrelated to selection into the included cohorts, differences in socioeconomic status and health consciousness between cohort participants and non-participants may limit the generalizability of our findings. It is also possible that our study may have been impacted by residual confounding by maternal health conditions such as latent gestational diabetes mellitus and hypertensive disorders, or supplement use, because women who are wealthier and more health conscious may use vitamin D supplements more often and also engage in other behaviors that impact offspring DNA methylation. It is also possible that our results may have been impacted by error in the measurement of vitamin D concentrations, as previous work has found that the quality of 25(OH)D measurement varies across cohorts, partly due to differing methods of assessment [57].

Our study was also limited by the measurement of DNA methylation within cord blood. While we did not find any strong associations between maternal vitamin D insufficiency, it is possible that maternal vitamin D insufficiency is more strongly associated with DNA methylation in other offspring tissue types, such as brain, bone, or respiratory tract tissues, though such tissues are much more difficult to obtain. Further work may be needed to evaluate whether our findings hold in different offspring tissue types and whether any such effects vary by fetal characteristics, including fetal sex. Similarly, our study relies on maternal vitamin D measurements at a single pregnancy time point, generally in mid-pregnancy. However, maternal vitamin D levels may change during pregnancy and impact methylation more strongly in early or late pregnancy. Importantly, our analysis may have been further limited by the restriction of the sample to the participants of White European ancestry to reduce confounding by race/ethnicity. While this restriction was necessary to reduce bias in the analysis, it is possible that the potential effects of vitamin D insufficiency on offspring methylation may be stronger in non-white participants, who are also more likely to experience more severe levels of vitamin D insufficiency [58]. This also limits the generalizability of these results to non-White women. Our results may also have been impacted by our choice to dichotomize vitamin D at clinical insufficiency levels. While this dichotomization may have limited the power of our analyses to detect epigenetic effects, the relationship between vitamin D and many health outcomes appears to be nonlinear [2,26,59], and our cut-off was selected based on clinical cut-offs relevant to medical decision making.

## Conclusions

We did not find strong evidence of an association between maternal mid-pregnancy vitamin D insufficiency and offspring cord blood methylation levels at any measured CpG site among white European ancestry mother–child pairs. Our results, consistent with a previous study of the topic, suggest that large, robust changes in neonatal DNA methylation in response to maternal vitamin D insufficiency are unlikely. However, it is possible that our study was limited by sample size, potential selection bias, or residual confounding. Future studies of the relationship between maternal vitamin D insufficiency and offspring DNA methylation could include more racial/ethnically diverse samples, larger sample sizes, and measurement of methylation in other offspring cell types and may consider exploring associations between offspring DNA methylation in cord blood and maternal vitamin D levels on offspring methylation at different periods across gestation.

## Supplementary Material

vitdewas_no_seasonality_BHpvalues20200703.zip

vitdewas_season_of_birth_BHpvalues20200703.zip

VitDEWAS_Supplementary Materials_Results_20240917.pdf

vitdewas_Supplementary Materials_20240726.docx

## Data Availability

The data supporting the results reported in this article can be found in the Supplementary Material. We are unable to make individual level data available due to concerns regarding compromising individual privacy. However, datasets of full meta-analysis results generated in this study are available in the Supplementary Material.
