# Supplementary material for: Epigenome-wide meta-analysis of prenatal vitamin D insufficiency and cord blood DNA methylation
Source: Epigenetics. 2024 Oct 17;19(1):2413815. doi: 10.1080/15592294.2024.2413815 (PMC11487971; doi:10.1080/15592294.2024.2413815)
Supplement: VitDEWAS_Supplementary Materials_Results_20240917.pdf [file KEPI_A_2413815_SM3531.pdf]

Supplementary Materials Part 1 – Maternal midpregnancy vitamin D EWAS

Table of Contents

1. Flowcharts describing probe inclusion for each model
2. Lambdas for base model for each participating cohort
3. Lambdas for season of birth model for each participating cohort
4. Lambdas for season of birth and folate model for each participating cohort
5. QQ Plots for base model for each participating cohort
6. QQ Plots for season of birth model for each participating cohort
7. QQ Plots for season of birth and folate model for each participating cohort
8. QQ Plots for full meta-analysis of base mode
9. PZ plots for base model for each participating cohort
10. PZ plots for season of birth model for each participating cohort
11. PZ plots for season of birth and folate model for each participating cohort

1.Flowcharts describing probe inclusion for each model

Base model & Season of birth model (no difference in exclusions)

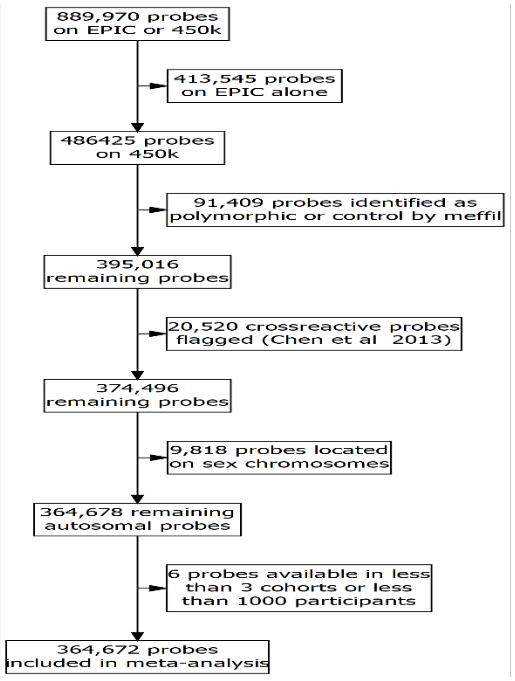

Full model

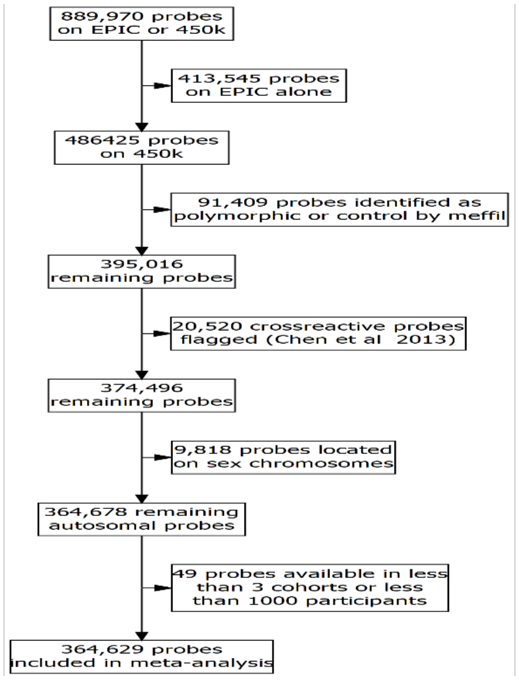

2.Lambdas for base model for each participating cohort

Cohort	n	Lambda
EAGeR	361	1.044
MoBa1	783	1.010
MoBa2	177	1.465
PREDO	301	0.850
Gen3G	175	1.321
Generation R	1154	1.033
Project Viva	283	0.929

3.Lambdas for season of birth model for each participating cohort

Cohort	n	Lambda
ALSPAC	499	0.981
EAGeR	361	1.047
MoBa1	783	0.861
MoBa2	177	1.157
PREDO	301	0.817
Gen3G	175	1.126
Generation R	1154	0.841
Project Viva	283	0.958

4.Lambdas for season of birth and folate adjusted model for each cohort

Cohort	n	Lambda
EAGeR	361	1.048
MoBa1	783	0.862
MoBa2	177	1.224
PREDO	301	0.866
Generation R	1154	0.814

5.QQ Plots for base model for each participating cohort

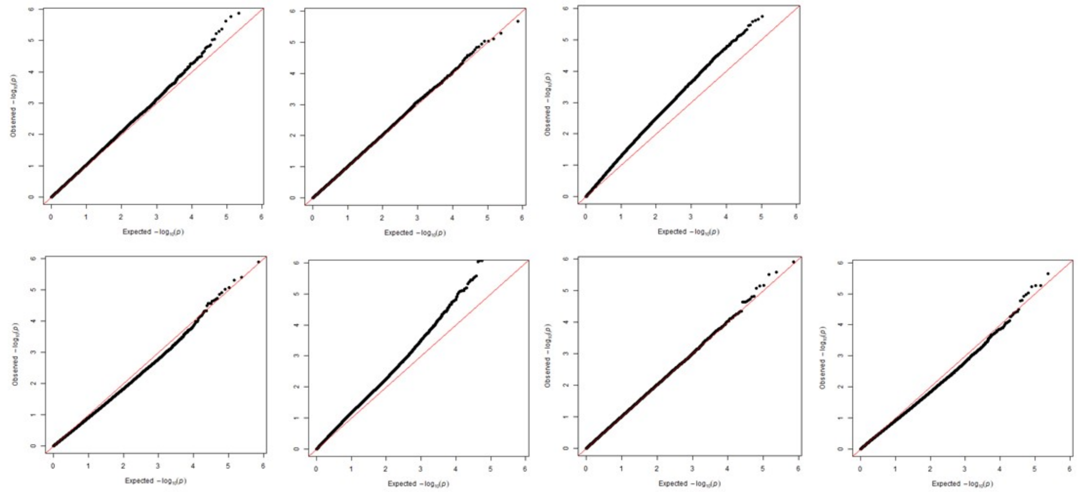

6.QQ Plots for season of birth model for each participating cohort

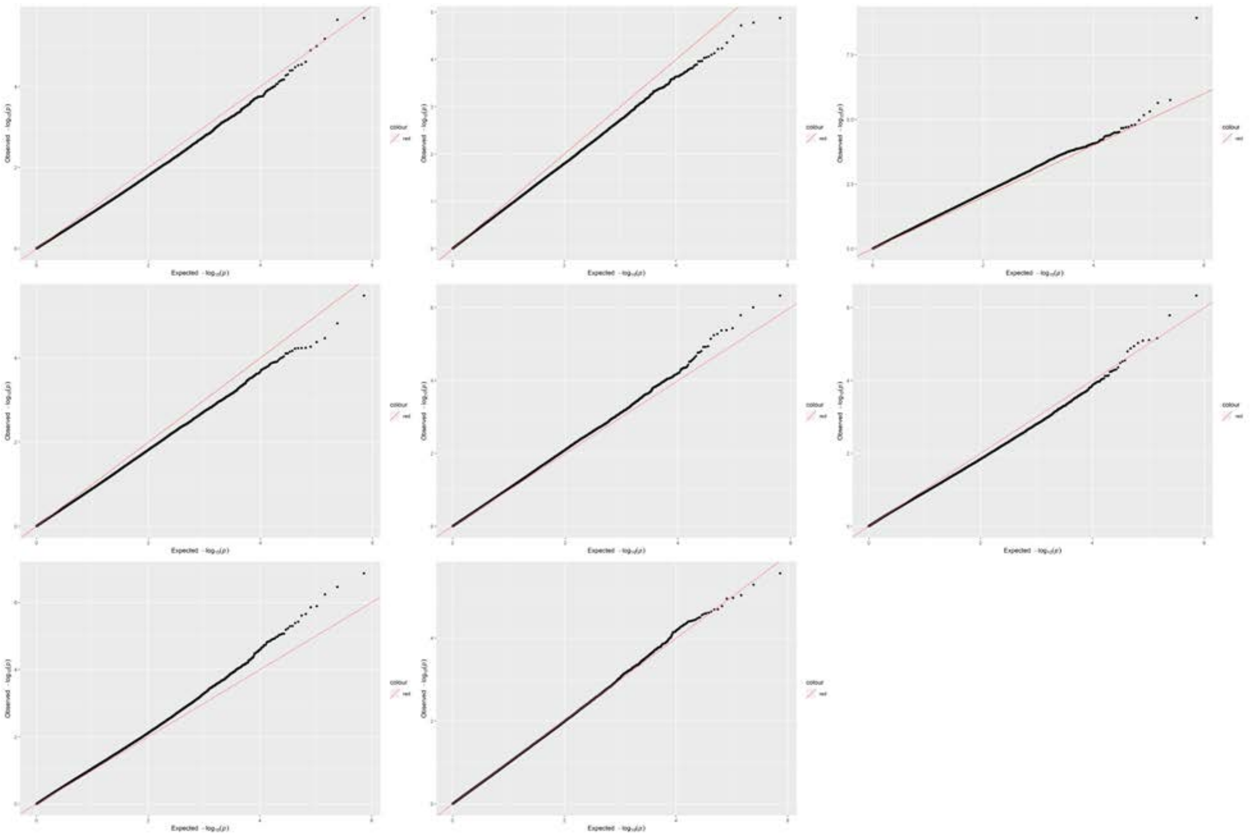

6.QQ Plots for season of birth and folate model for each participating cohort

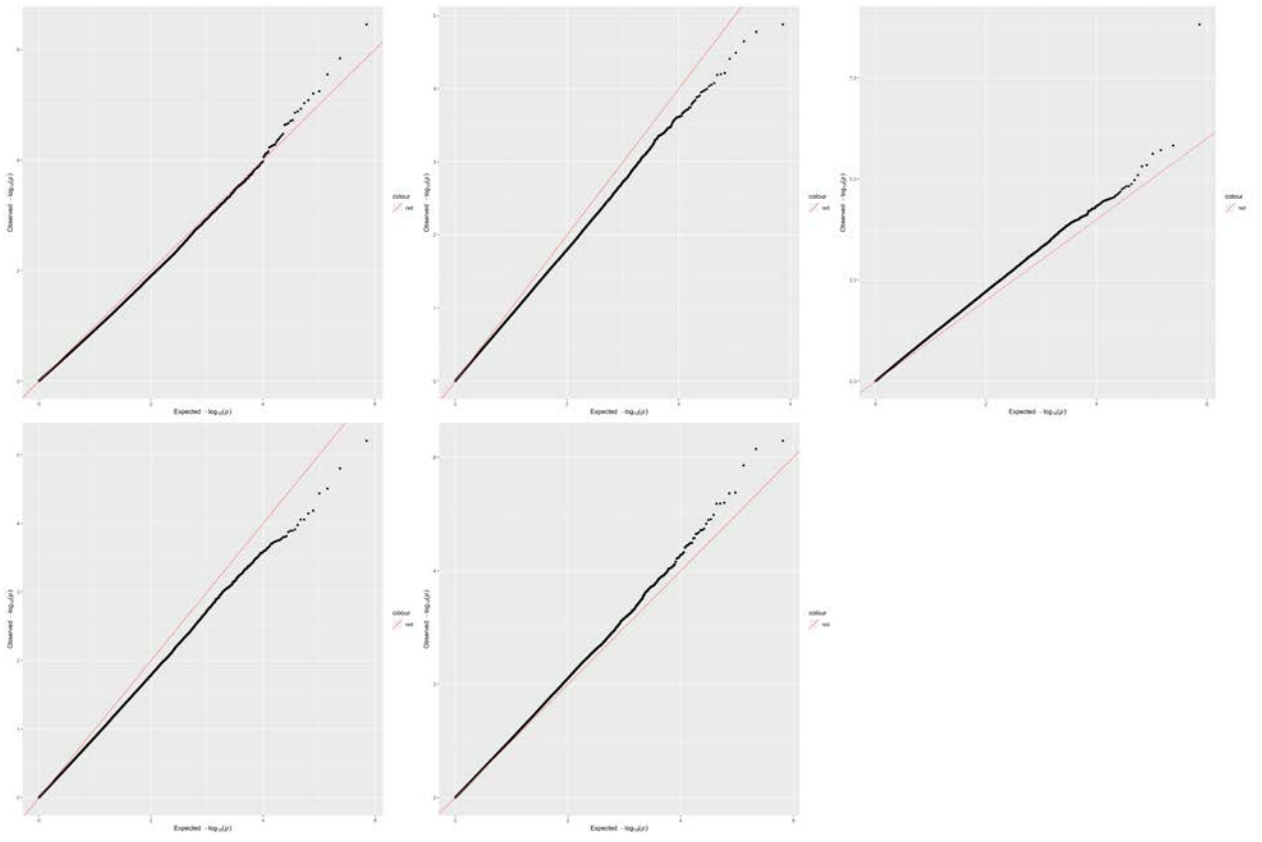

7.QQ Plots for full meta-analysis results

Base Model

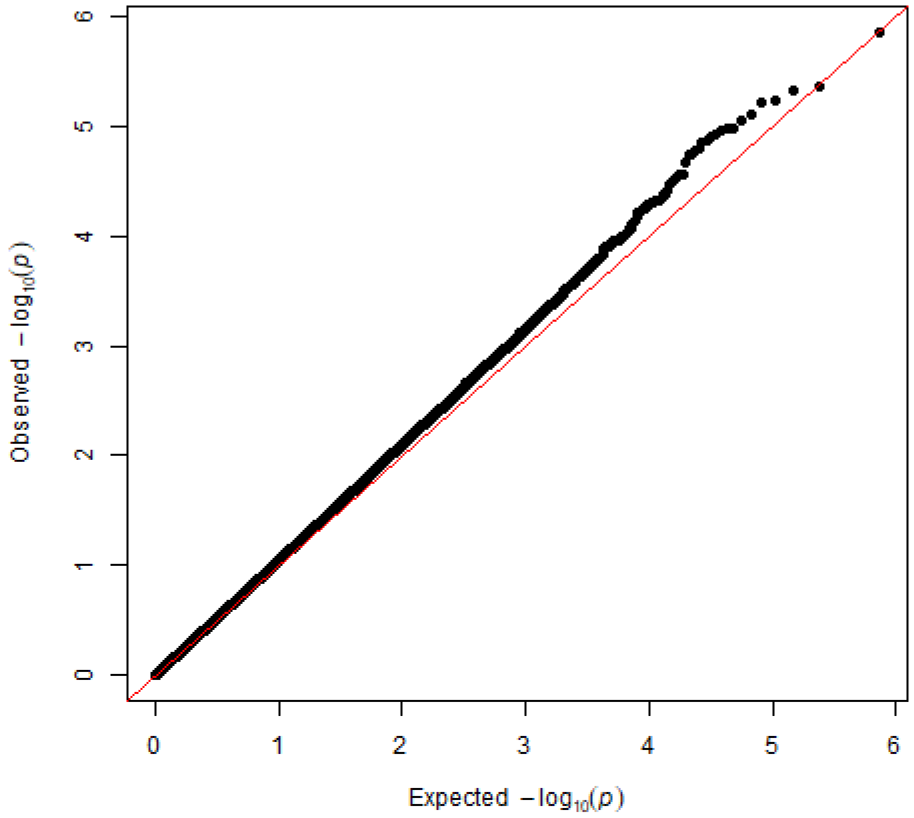

Season of Birth Model

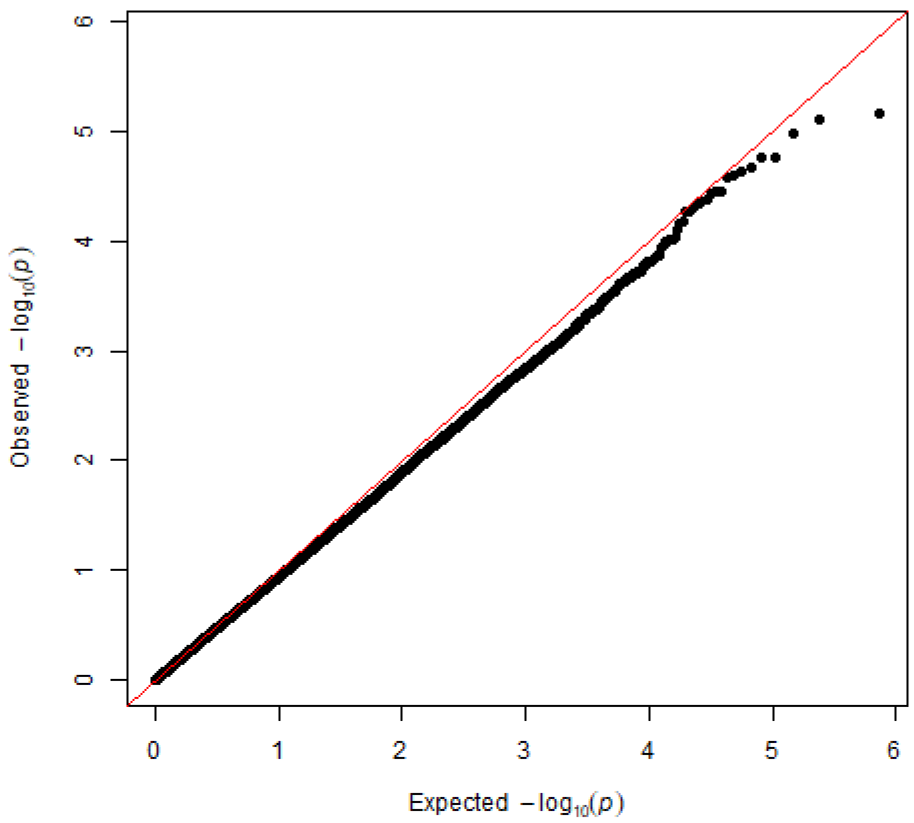

Full Model

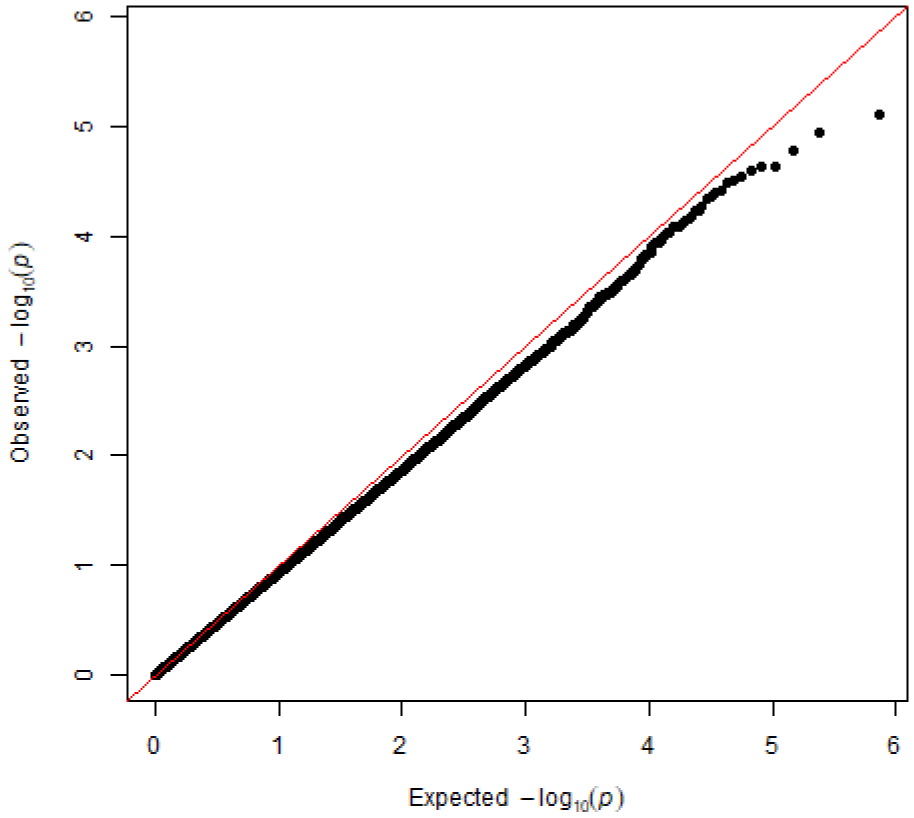

7.PZ plots for base model for each participating cohort

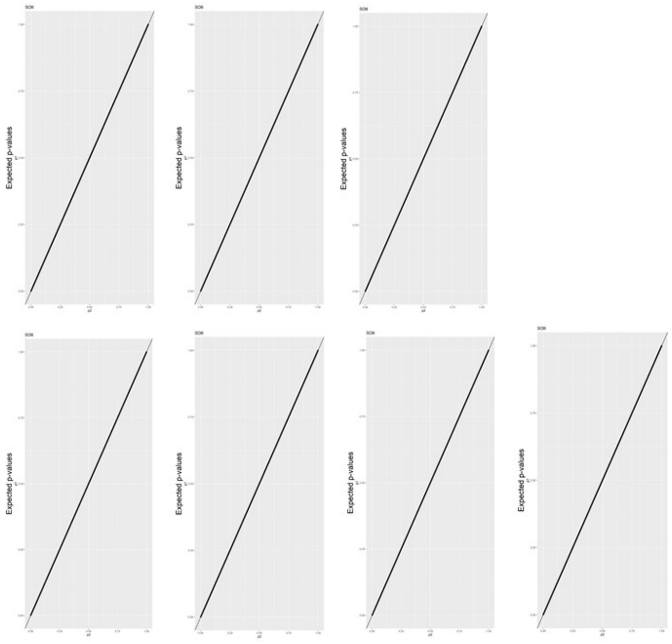

8.PZ Plots for season of birth model for each participating cohort

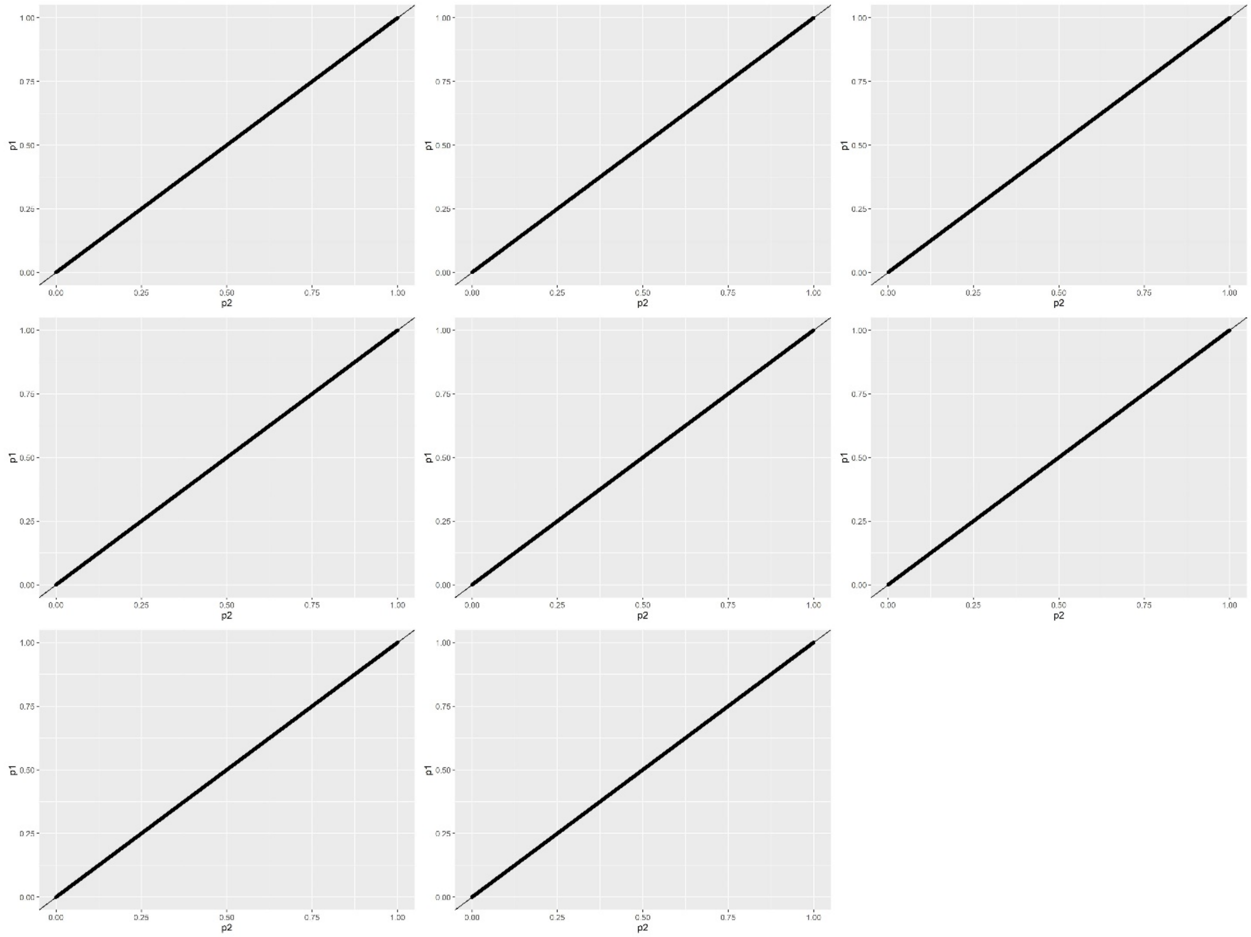

9.PZ Plots for season of birth and folate model for each participating cohort

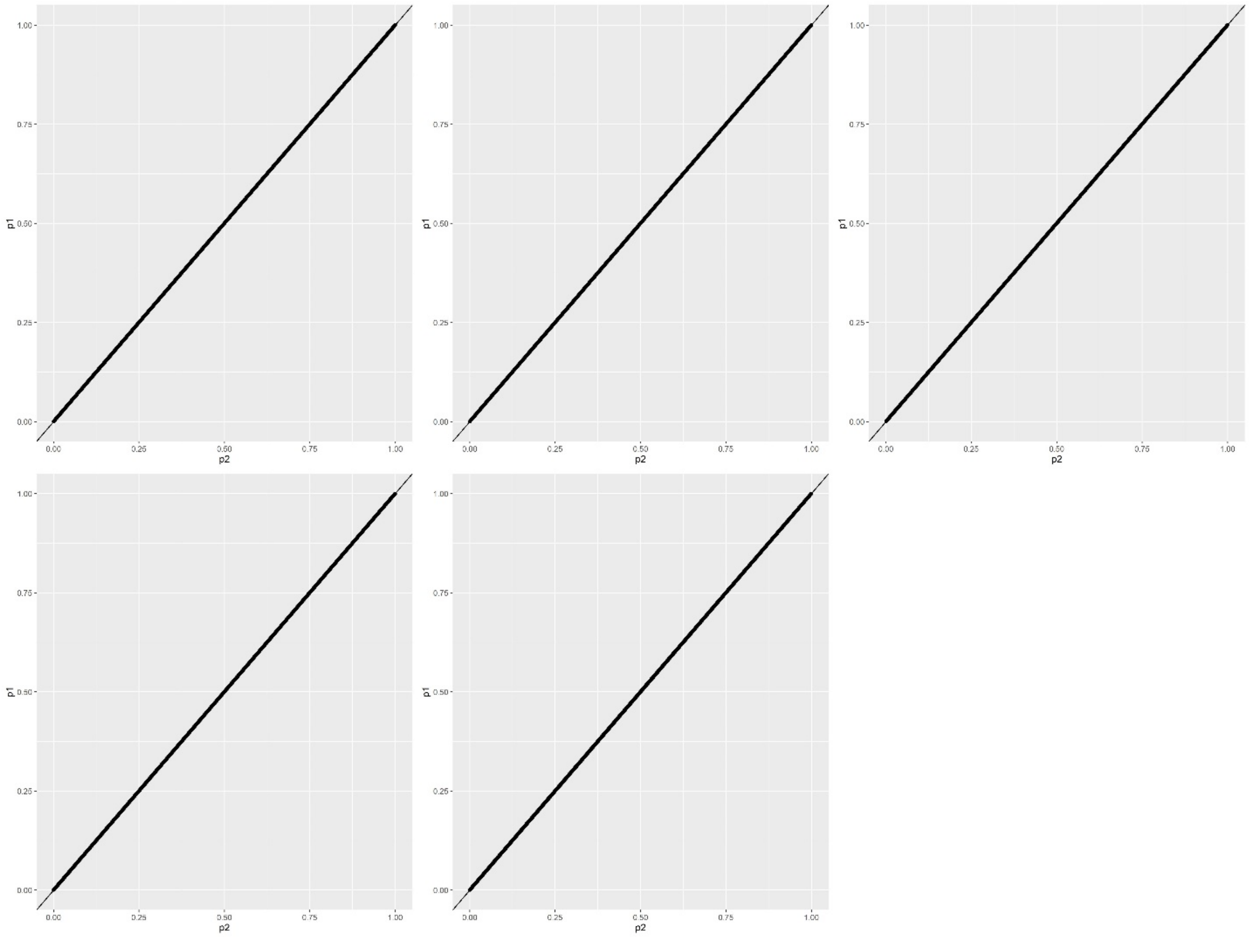

10. Boxplots of betas for each cohort (base model)

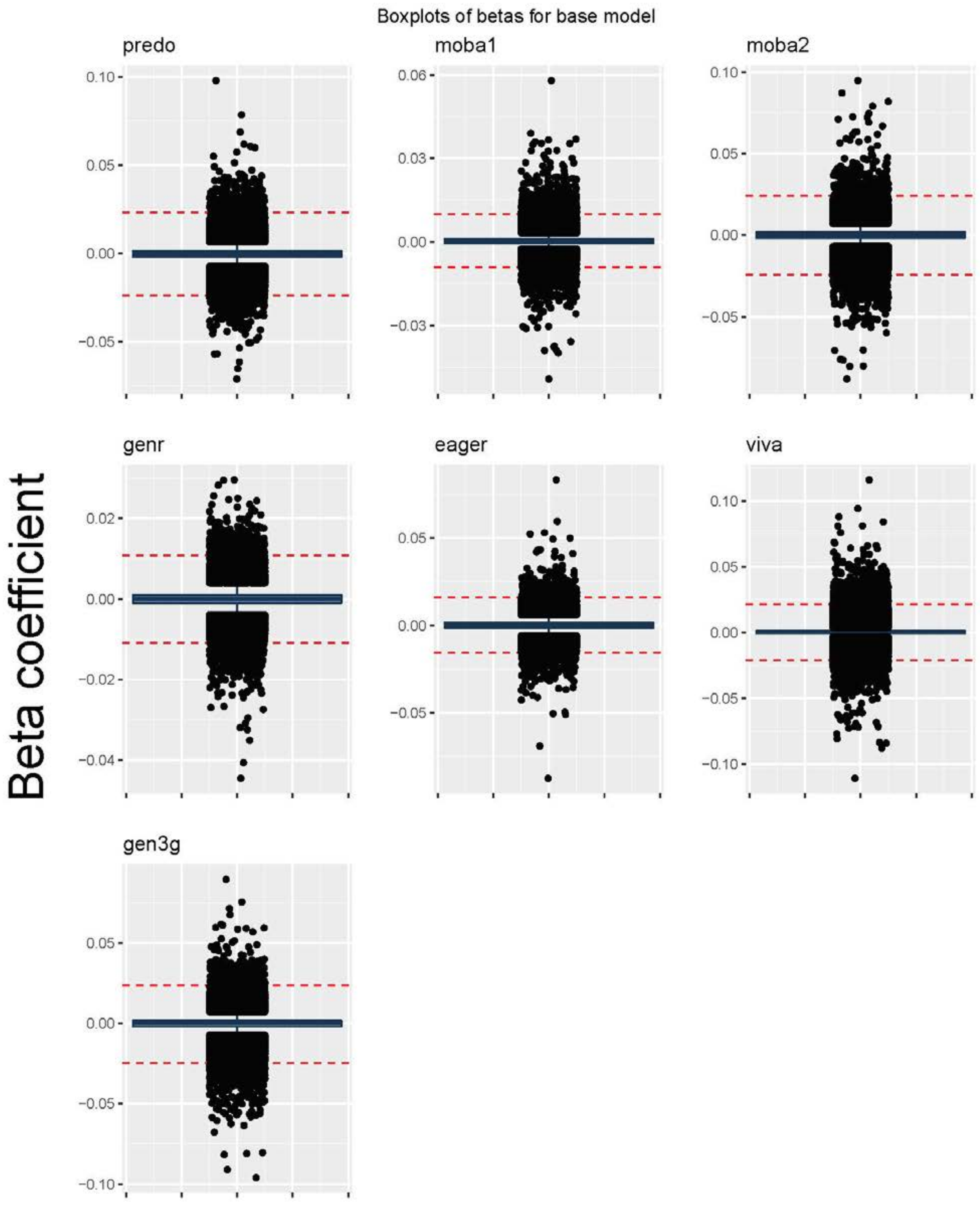

Red dashed lines correspond to 5 standard deviations.

11.Boxplots of betas for each cohort (season of birth model)

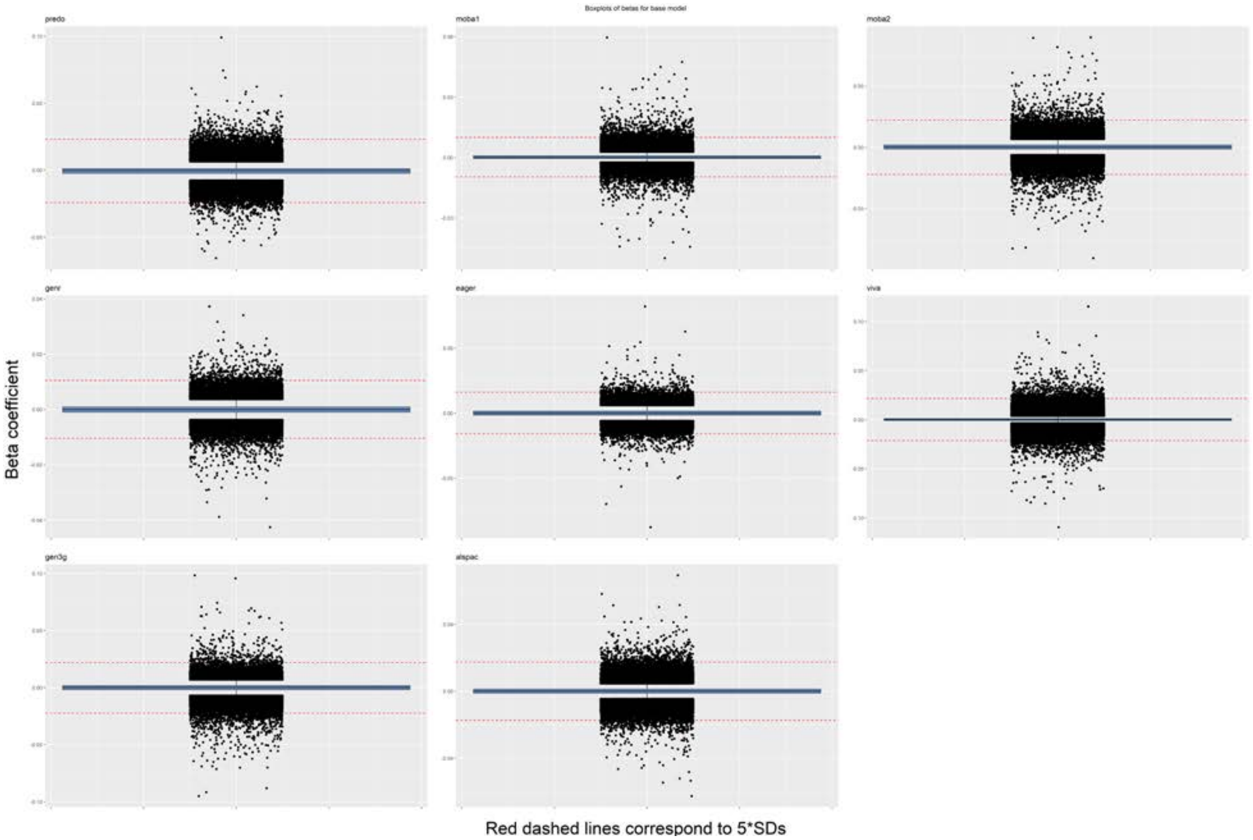

12. Boxplots of betas for each cohort (season of birth and folate model)

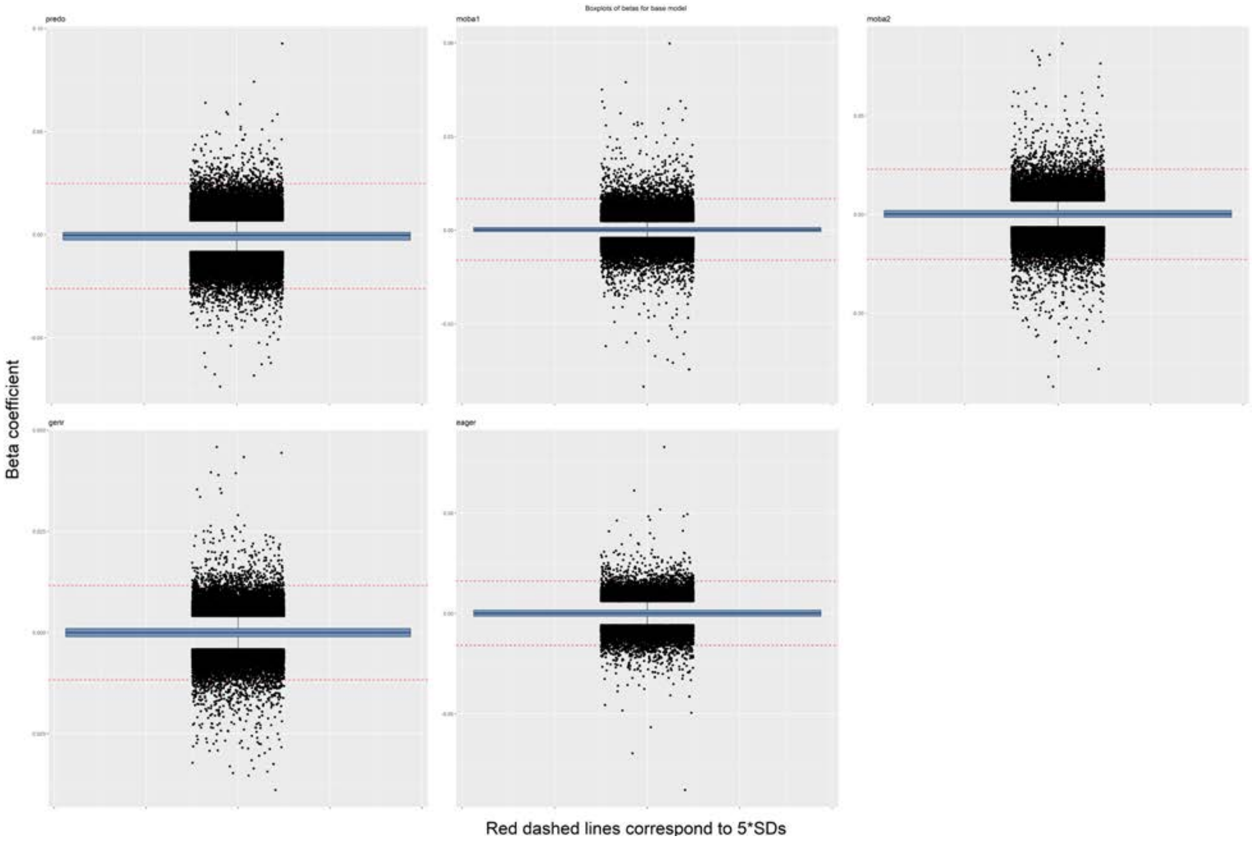

13. Plots of precision relative to sample size (base model)

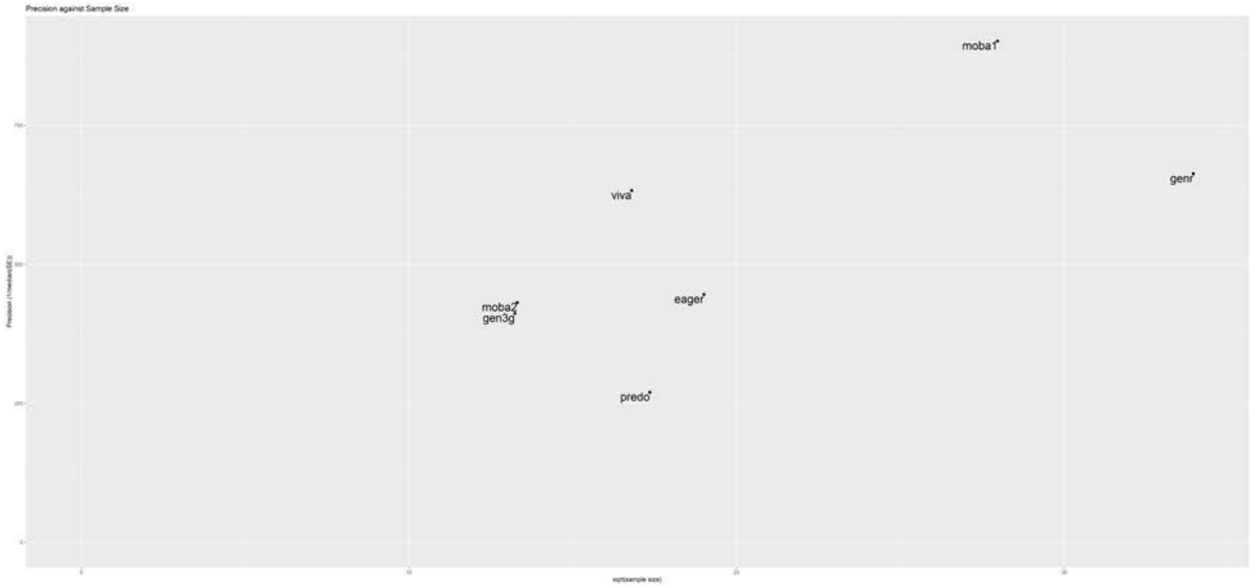

14. Plots of precision relative to sample size (season of birth model)

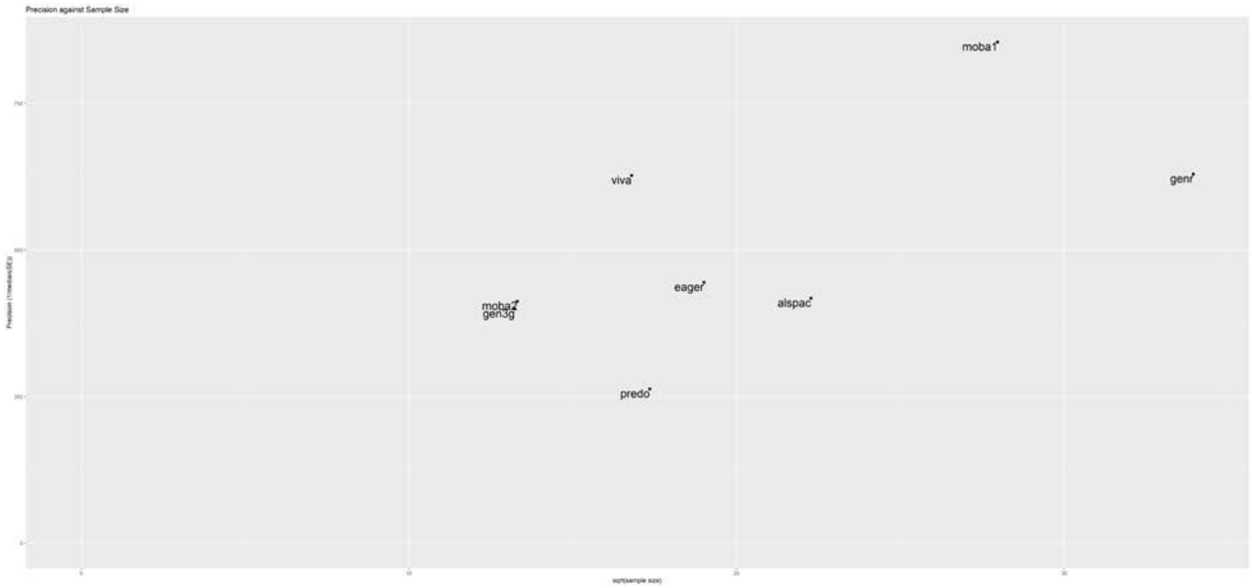

15. Plots of precision relative to sample size (season of birth and folate model)

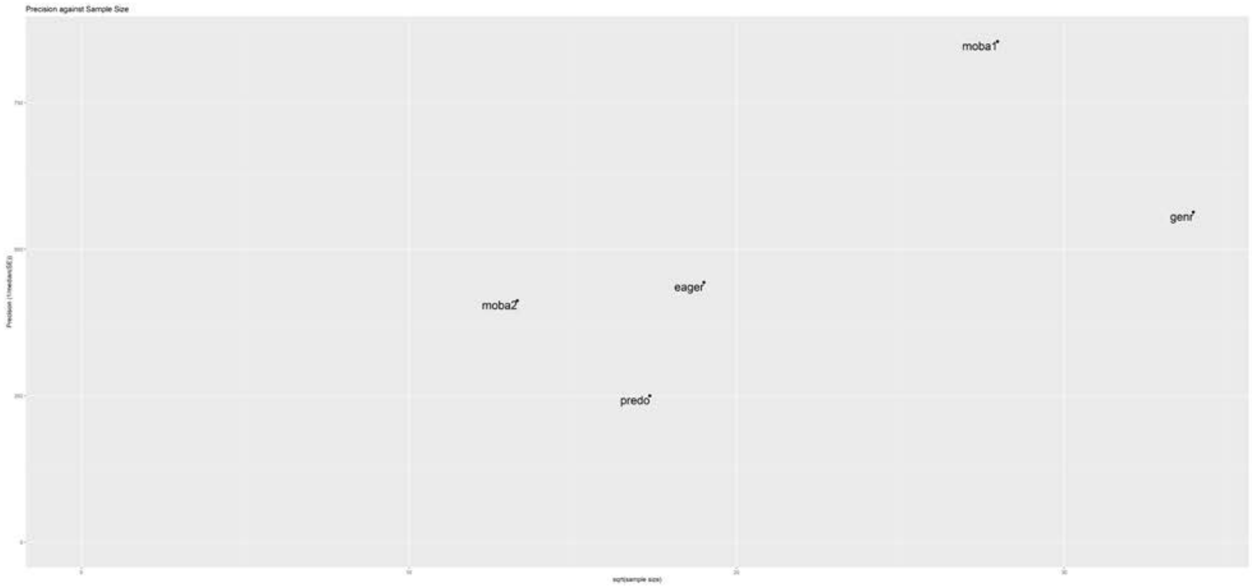

16. Volcano plots for each cohort (base model)

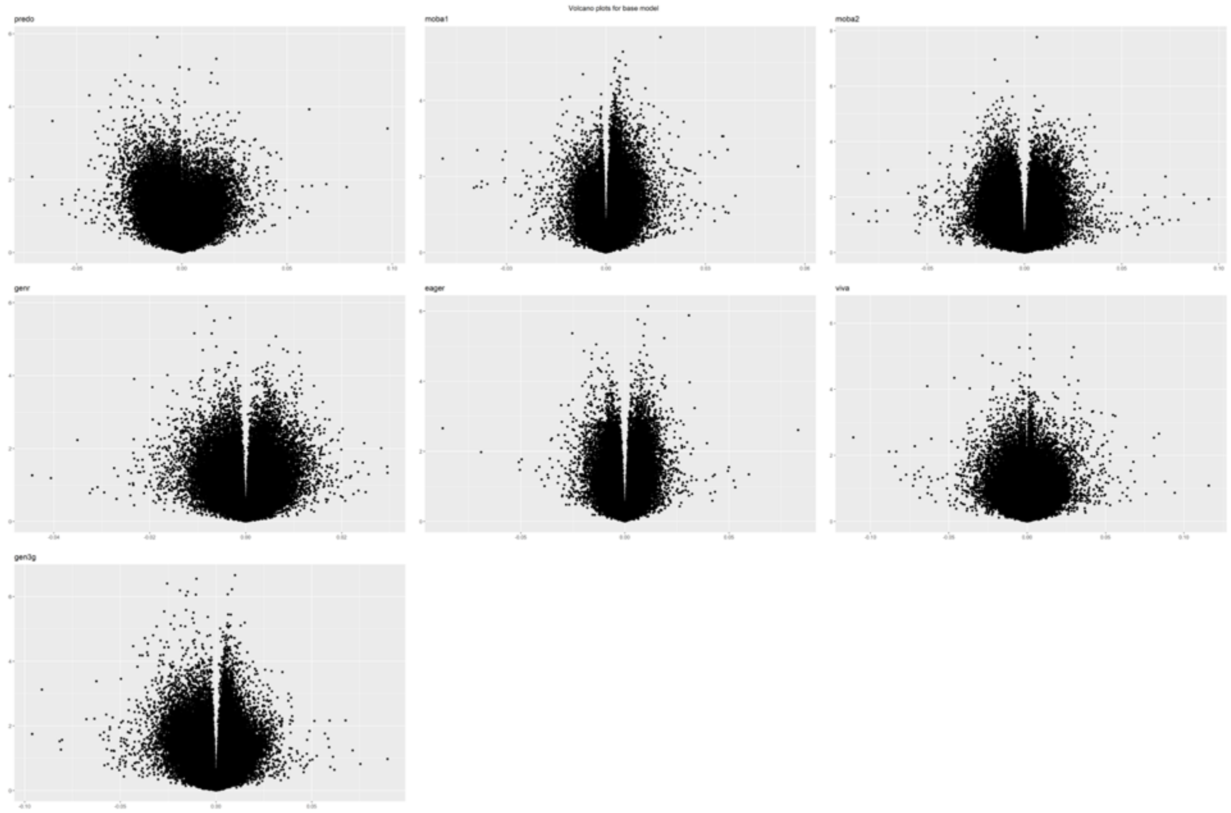

17. Volcano Plots for each cohort (season of birth model)

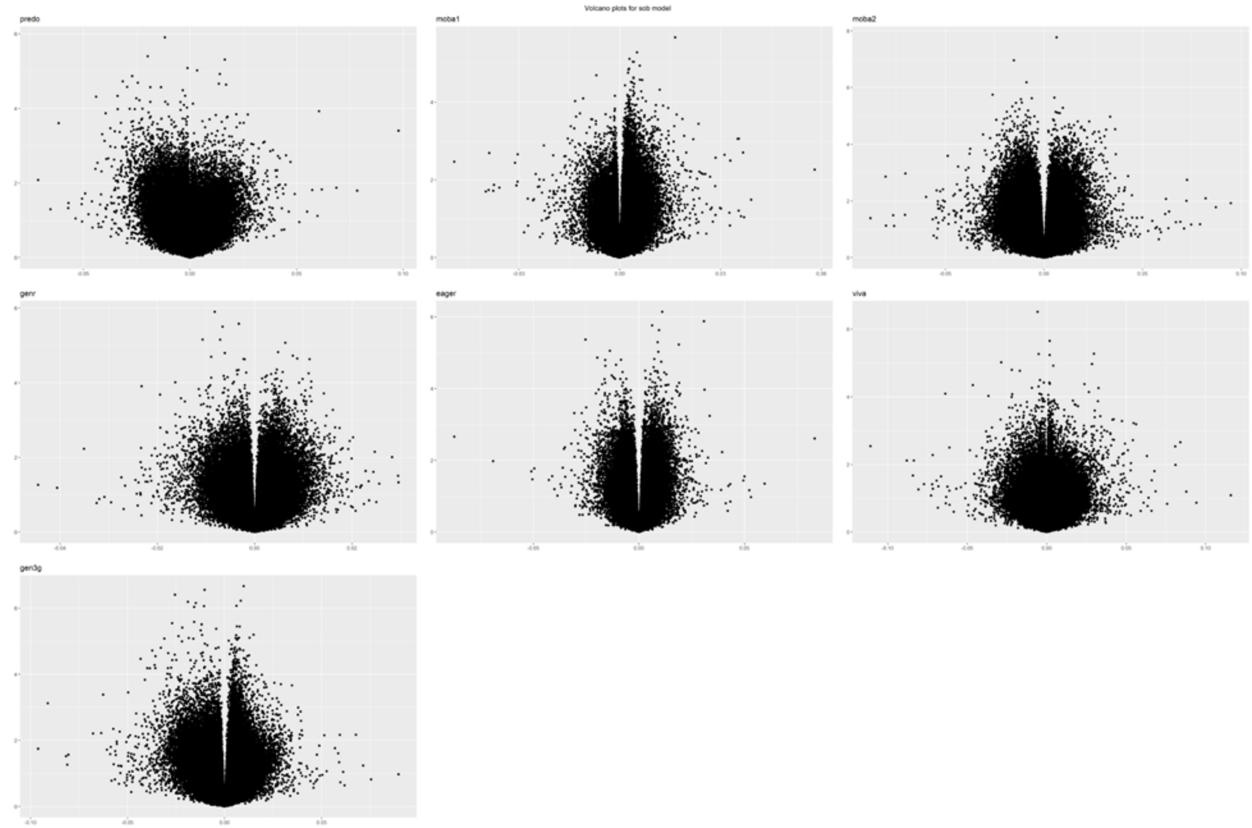

18. Volcano Plots for each cohort (season of birth and folate model)

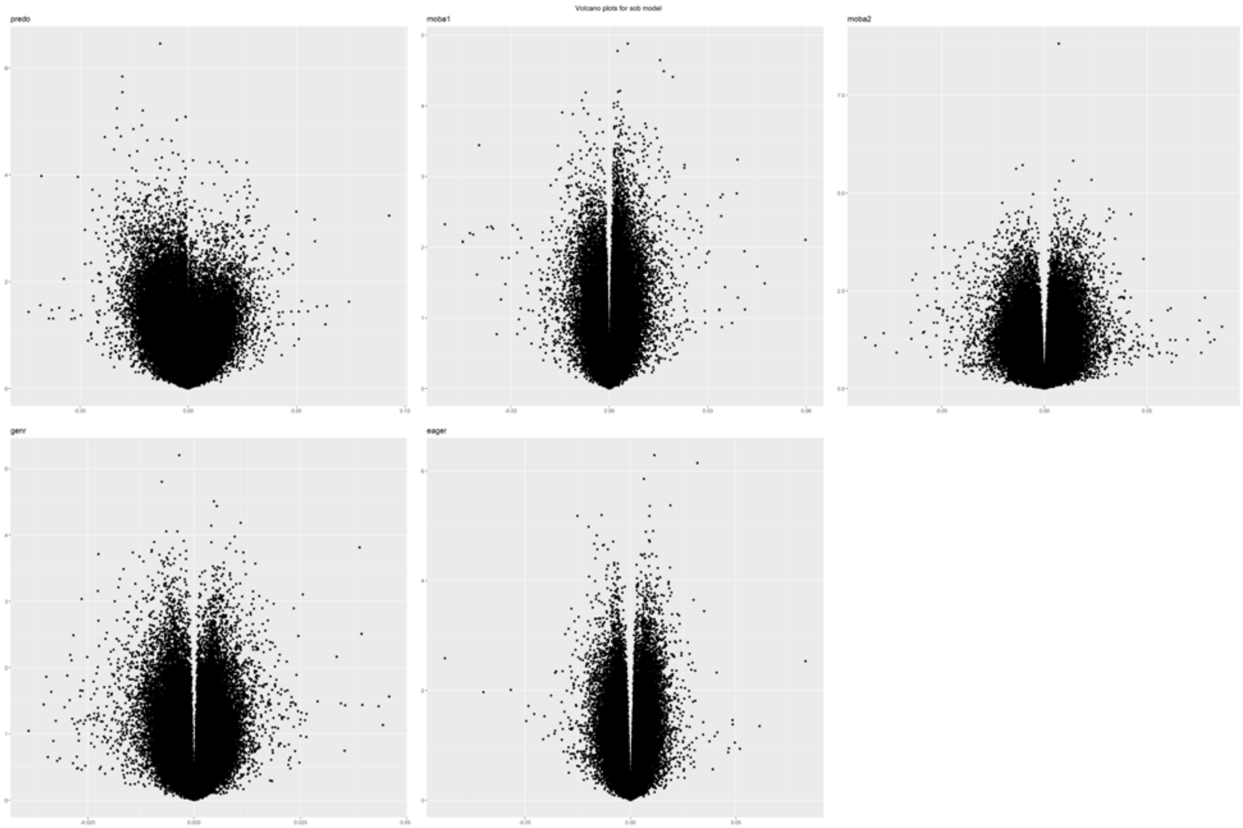
Volcano plots for sob model
predo
moba1
moba2
genr
eager
