# Supplementary material for: Epigenome-wide meta-analysis of prenatal vitamin D insufficiency and cord blood DNA methylation
Source: Epigenetics. 2024 Oct 17;19(1):2413815. doi: 10.1080/15592294.2024.2413815 (PMC11487971; doi:10.1080/15592294.2024.2413815)
Supplement: vitdewas_Supplementary Materials_20240726.docx [file KEPI_A_2413815_SM3530.docx]

Supplementary Materials

Cohort-specific descriptions of data collection

**ALSPAC**

*Study population*

ALSPAC is a “transgenerational prospective observational study investigating influences on health and development across the life course”[1, 2]. Pregnant women resident in Avon, UK with expected dates of delivery 1st April 1991 to 31st December 1992 were invited to take part in the study. A total of 14,541 pregnancies were initially enrolled. From these pregnancies, there were a total of 14,676 fetuses, resulting in 14,062 live births and 13,988 children who were alive at 1 year of age. Detailed information has been collected on these women and their offspring at regular intervals. In addition, at age 7, an attempt was made to recruit additional eligible cases. Therefore, from age 7 onwards, the sample includes data on 15,454 pregnancies, resulting in 15,589 foetuses. The study website contains details of all the data that is available through a fully searchable data dictionary ([http://www.bristol.ac.uk/alspac/researchers/our-data/](https://eur01.safelinks.protection.outlook.com/?url=http%3A%2F%2Fwww.bristol.ac.uk%2Falspac%2Fresearchers%2Four-data%2F&data=04%7C01%7Ce.diemer%40erasmusmc.nl%7C6f5a4f08f86b4c13908908d90eedda82%7C526638ba6af34b0fa532a1a511f4ac80%7C0%7C1%7C637557237027779804%7CUnknown%7CTWFpbGZsb3d8eyJWIjoiMC4wLjAwMDAiLCJQIjoiV2luMzIiLCJBTiI6Ik1haWwiLCJXVCI6Mn0%3D%7C2000&sdata=hbAFluYRG%2F110%2FHRS1ZaPG4ExOA%2B1M8rv22cgGJA9lc%3D&reserved=0) ). DNA methylation was measured in cord blood for approximately 1000 mother-child pairs. For the current study, we restricted analyses to pairs with complete data on mid-pregnancy Vitamin D, offspring cord blood methylation, and all covariates, resulting in a total analytic sample of 499.

*Maternal mid-pregnancy Vitamin D*

25(OH)D concentrations were measured in serum of non-fasting blood samples taken as part of antenatal care. Samples could be taken from any stage of pregnancy. Measurements were made using high-performance liquid chromatography tandem mass spectrometry in one laboratory. Since vitamin D levels may change with time of the year, and pregnant women with length of gestation, measurements were simultaneously adjusted for both factors to obtain an estimate at 28 weeks gestation [3]. Other estimates to 0 and 34 weeks gestation were highly correlated (R ~ 0.6 and 0.9, respectively).

*Offspring cord blood DNA methylation*

DNA methylation was measured for approximately 1000 mother-child pairs in the cord blood and peripheral blood of study children at ages 7 and 15-17 years and in the peripheral blood of mothers approximately 18 years after the birth of the study child. The resulting profiles comprise the Accessible Resource for Integrated Epigenomics Studies [4](ARIES, http://www.ariesepigenomics.org.uk/). All data are available by request from the Avon Longitudinal Study of Parents and Children Executive Committee (http://www.bristol.ac.uk/alspac/researchers/access/) for researchers who meet the criteria for access to confidential data. Ethical approval for the study was obtained from the ALSPAC Ethics and Law Committee and the Local Research Ethics Committees. Consent for biological samples has been collected in accordance with the Human Tissue Act (2004).

Genomic DNA was obtained from blood samples and bisulphite converted using the Zymo EZ DNA MethylationTM kit (Zymo, Irvine, CA). DNA methylation was quantified using the Illumina HumanMethylation450 BeadChip according to manufacturer’s instructions. During the data generation process a wide range of batch, variables were recorded in a purpose-built laboratory information management system (LIMS) [2].The LIMS also reported quality control (QC) metrics from the standard control probes on the 450k BeadChip for each sample. Samples failing QC were excluded from further analysis and the assay repeated. Sample QC and normalization was completed using the meffil package as previously described [5]. Briefly, probe intensities underwent a functional normalization approach [6]using the first 10 PCs of the Illumina 450K array control probes. This approach includes subset quantile normalization of the data and normal-exponential out-of-band background correction. In addition, twenty surrogate variables [7] were generated and included in all regression models.

*Covariates*

Maternal smoking status, pre-pregnancy weight, maternal education, maternal age, and parity were obtained by questionnaire during pregnancy. Information on fetal sex was obtained via self report and administrative records. Estimation of six different white blood cell types (CD8+ T and CD4+ T lymphocytes, CD56+ natural killer cells, CD19+ B cells, CD14+ monocytes, and granulocytes) by Houseman method[15].

**EAGeR**

*Study Population*

The Effects of Aspirin in Gestation and Reproduction (EAGeR) trial was a block-randomized, double-blind, placebo-controlled trial evaluating the effect of preconception-initiated daily low dose aspirin on live birth. Details of the trial design have been described in detail elsewhere[8]. Briefly, participants who had previously experienced 1-2 prior pregnancy losses, and were currently attempting to conceive were recruited from 2007 to 2011 at 4 United States medical centers. Exclusion criteria for the study included a known history of infertility treatment, pelvic inflammatory disease, tubal occlusion, endometriosis, anovulation and polycystic ovarian syndrome, or uterine abnormality, resulting in enrollment of 1,228 women. Participants were followed for six menstrual cycles while attempting pregnancy or throughout pregnancy if they conceived. For the current study, we restricted the sample to women who had conceived, and had complete data on midpregnancy vitamin D, offspring cord blood methylation, and all covariates, resulting in an analytic sample of 361 mother-child pairs.

*Maternal midpregnancy Vitamin D*

Serum samples were collected at 8 weeks gestation and cryostored at -80° C prior to analysis. Total 25-hydroxy-vitamin D [25(OH)D] was measured in serum with the 25-hydroxyvitamin D ELISA solid phase sandwich enzyme immunoassay (BioVendor R&D, Ashville, NC, USA). Further details on the measurement of 25(OH)D in the EAGeR trial have been published previously [9].

*Offspring Cord Blood Methylation*

Beginning in 2009, the trial collected 10 ml cord blood from over 90% of deliveries at the Utah trial site. Cord blood was centrifuged and separated into plasma and buffy coat. Samples were subsequently frozen at -80 degrees C. Genome-wide DNA methylation was measured with the Infinium MethylationEPIC Bead Chip. Methylation data were processed using the minfi package in R, which included identification of failed probes and scaling with Illumina control probes to determine methylation values. Quantile normalization was used to normalize beta values between two types of probes. We used principal component analysis (PCA) to detect further outliers and samples mismatched for sex. Samples mismatched for sex were excluded. Beta values were replaced as missing if the detection P-value was > 0.01 or bead counts <3. In the analyses the results were set as "NA", when the missing values of the CpG site is greater than 3% to enable the code to run without the need to remove the missing values.

*Covariates*

Cell type mixture was estimated on the full set of normalized methylation data using the Bakulski et al., 2016 reference dataset for cord blood (FlowSorted.CordBlood.450K package)[10]. Maternal age, smoking status, and income were measured via self-report. Maternal pre-pregnancy BMI was measured directly, prior to pregnancy.

**Gen3G**

*Study Population*

The Genetics of Glucose regulation in Gestation and Growth (Gen3G) cohort is a prospective observational pre-birth cohort study aimed at investigating glucose regulation determinants in pregnancy and fetal growth, based on the Eastern Townships Region of Quebec, Canada[11]. All women who received prenatal care directly at the Centre Hospitalier Universitaire de Sherbrooke (CHUS), a CHUS affiliated health center, or planned delivery at CHUS between January 2010 and June 2013, were considered eligible. A total of 1034 pregnant were women were recruited at the 1^st^ trimester, of which 10 were excluded due to the presence of a multiple pregnancy. Additional exclusion criteria included known pre-pregnancy diabetes, use of a medication known to influence glucose tolerance, glycated haemoglobin (HBA1c) >= 6.5% of 1 h glucose >= 10.3 mmol/L post 50 g glucose challenge test, miscarriage, medical abortions, or health problems that prohibited participation. A total of 854 participants were followed through delivery. Recruitment and characteristics of the cohort have been described in detail elsewhere [11]. For the current study, we restricted to mother-child pairs with complete data on maternal mid-pregnancy Vitamin D, offspring DNA methylation, and available covariates, resulting in a total analytic sample of 175 pairs.

*Maternal mid-pregnancy Vitamin D*

Non-fasting blood samples were collected at the first study visit (mean 9.6 weeks gestation). Aprotinin was added to blood samples, which were centrifuged at 2500g at 4 degrees C for 10 minutes, and aliquoted for storage at -80 degrees C. 25(OH)D_2_ and 25(OH)D_3_ concentrations were assessed using liquid-liquid extraction followed by liquid chromatography-electrospray tandem mass spectrometry (Quattro micro mass spectrometer; Waters, Milford, MA). 25(OH)D concentrations were calculated as the total of the two measurements[12].

*Offspring DNA methylation*

Cord blood samples were collected via syringe from the umbilical vein after delivery. Bisulfite conversion was performed using the EZ-96 DNA methylation kit (Zymo research Corporation, Irvine, USA). We used the Infinium HumanMethylation450 BeadChip (Illumina Inc., San Diego, USA) to measure the methylation level as a beta value ranging from 0 (no methylation) to 1 (complete methylation). During quality control, we removed samples that were outliers on the MDS plot, samples with > 5% missingness, probes missing in more than 20% of samples, and duplicates. DASEN normalization was performed using the watermelon package in R. In analyses, ComBat was used to adjust for sample Plate while protecting dichotomized vitamin D in the model statement.

*Covariates*

Fetal sex was collected from medical records. Maternal age and parity were assessed via questionnaire at the beginning of pregnancy. Gestational age was calculated based on reported last menstrual period, and corrected by ultrasound dating when appropriate. Maternal smoking self-reported in the first trimester questionnaire, and grouped into 3 categories (no smoking in pregnancy, stopped smoking in the beginning of pregnancy, and smoked during pregnancy). Maternal early pregnancy BMI was calculated using weight and height measured by research staff according to standard procedures at the first trimester visit. Measures of maternal education were not available in this cohort. The sample included in this analysis is comprised entirely on women of European ancestry. We used the Bakulski -based Houseman method[13, 14] with the estimate Cell Counts function in the Minfi package[15] in R[16] to estimate relative proportions of six white blood cell subtypes (CD4+ T-lymphocytes, CD8+ T-lymphocytes, natural killer (NK) cells, B-lymphocytes, monocytes and granulocytes).

**Generation R Study**

*Study Population*

The GenerationR Study is a prospective birth cohort from fetal life to young adulthood, based in Rotterdam, the Netherlands [17, 18]. Pregnant women who lived in the Rotterdam area and had a delivery date between April 2002 and January 2006 were recruited by participating midwives and obstetricians. While the study aimed to recruit women during early pregnancy, women were allowed to enroll at any point during pregnancy, or in the first months after birth during routine visits to child health centers. In total 9,778 mothers were enrolled, 8,880 of whom were enrolled during pregnancy. Recruitment and characteristics of the cohort have been described in detail elsewhere [17, 18]. Participants were only eligible for methylation analysis if they were additionally part of the Generation R Focus cohort, a subset of the study selected characterized by Dutch ethnicity and a high level of completeness of collected data. For the current study, we restricted the sample to mother –child pairs with complete data on maternal mid-pregnancy vitamin D, offspring DNA methylation, and all covariates, resulting in an analytic sample of 1,154 mother-child pairs.

*Maternal mid-pregnancy Vitamin D*

Maternal vitamin D concentrations were measured in serum samples taken in weeks 18.1-24.9 of gestation. Details of the collection procedure have been described in detail elsewhere [19]. Briefly, 50 μL milli-Q water and 50 μL of acetonitrile (ACN) containing 6,19,19-[2H3]-25OHD2 and 6,19,19-[2H3]-25OHD3 at 10 nmol/L each were added to 3 μL plasma, sonicated, vortexed and centrifuged. The supernatant was filtered using a TiO2/ZrO2 filter plate (Glygen, USA) and evaporated to dryness. Samples were derivatised using 4-phenyl-1,2,4-triazoline-3,5-dione (PTAD) and reconstituted in ACN:H2O (1:3) prior to analysis. Samples were quantified using isotope dilution liquid chromatography-tandem mass spectrometry. The analytical system was comprised of a Shimadzu Nexera UPLC coupled to an AbSciex 5500 QTRAP equipped with an APCI source. Chromatographic separation was achieved using a Kinetex XB-C18 column (50 × 2.1 mm, 1.7 μm; Phenomenex, USA), and 72% acetonitrile/32% aqueous 0.1% formic acid at a flow rate of 0.5 mL/min. Total Serum 25(OH)D was calculated as the sum of Serum 25(OH)D_2_ and 25(OH)D_3_.

*Offspring DNA methylation*

Directly after delivery, obstetricians and midwives collected a maximum of 30 ml cord blood from the umbilical vein. DNA extraction from all children using the Qiagen Flexigene Kit (Qiagen Hilden, Germany)[20]. In a subgroup of 1339 Generation R children of Dutch ancestry, 500 ng DNA per sample underwent bisulfite conversion using the EZ-96 DNA Methylation kit (Shallow) (Zymo Research Corporation, Irvine, USA). Samples were plated onto 96-well plates in no specific order. Samples were processed with the Illumina Infinium HumanMethylation450 BeadChip (Illumina Inc., San Diego, USA), which analyses methylation at 485,577 CpG sites. Preparation and normalization of the HumanMethylation450 BeadChip array data was performed according to the CPACOR workflow1 using the software package R2. In detail, the idat files were read using the minfi package. Probes that had a detection p-value above background (based on sum of methylated and unmethylated intensity values) ≥ 1E-16 were set to missing per array. Next, the intensity values were stratified by autosomal and non-autosomal probes and quantile normalized for each of the six probe type categories separately: type II red/green, type I methylated red/green and type I unmethylated red/green. Beta values were calculated as proportion of methylated intensity value on the sum of methylated+unmethylated+100 intensities. Arrays with observed technical problems such as failed bisulfite conversion, hybridization or extension, as well as arrays with a mismatch between sex of the proband and sex determined by the chr X and Y probe intensities were removed from subsequent analyses. Additionally, only arrays with a call rate > 95% per sample were processed further. Probes on the X and Y chromosomes were excluded from the dataset. The final dataset contained information on 458,563 CpGs.

*Covariates*

Fetal sex, maternal smoking (categorized as no smoking during pregnancy, stopped smoking in early pregnancy, or continued smoking in pregnancy), maternal age at birth, maternal education (categorized as no education, primary education, secondary education (phase 1), secondary education (phase 2), higher education (phase 1), or higher education (phase 2)) , parity, and pre-pregnancy BMI were assessed via self-report questionnaire during pregnancy. Cell type correction was applied using the reference-based Houseman method3 in the minfi package4 in R2, using the cord blood-specific Bakulski reference [10]. Because the subsample of Generation R data with available methylation data was entirely of European ancestry, principal components were calculated using only Generation R children of European ancestry, and the first 10 principal components were included as covariates in the models.

**MoBa**

*Study population*

The Norwegian Mother, Father, and Child Study (MoBa) is a prospective population based birth cohort conducted by the Norwegian Institute of Public Health. Between 1999 and 2008, all pregnant women in Norway were invited to take part in MoBa via postal invitation distributed after their routine ultrasound examination at 17-18 week’s gestation, of which 40.6% agreed to participate. Detailed information is available elsewhere [21]. The cohort contains linked data on 114,500 children, 95,200 mothers, and 75,200 fathers. MoBa1 and MoBa2 are two subsets of the total MoBa sample on which methylation data were obtained. MoBa1 consists of a case-cohort sample of 1,068 randomly drawn MoBa children born between July 2002 and July 2003, along with all MoBa children born between July 2002 and July 2004 whose mother reported they had received a diagnosis of asthma by age 3 and were using an inhalation medication for asthma at age 3, and who had remained in the study through age 3 [22]. MoBa2 consisted of a second case-control sample of 685 MoBa children, of which a third had diagnosed asthma by age 7, with complete data on maternal plasma folate during pregnancy [23]. The two samples were analyzed separately. For the current analysis, we restricted samples to individuals with complete data on maternal vitamin D during pregnancy, offspring methylation, and all covariates, resulting in analytic samples of 783 and 177 for MoBa1 and MoBa2, respectively.

*Maternal Mid-pregnancy Vitamin D*

25OHD concentrations were measured in plasma from blood samples taken at the 17^th^-18^th^ week of pregnancy[24]. Maternal plasma levels of 25-hydroxyvitamin D_3_ and 25-hydroxyvitamin D_2_ were analyzed using a liquid chromatography-tandem mass spectrometry method (LC–MS/MS) at the BEVITAL laboratory. BEVITAL is approved by the Vitamin D External Quality Assurance Scheme.  The sum of 25-hydroxyvitamin D_3_ and -D_2_, termed 25(OH)D, was used in the analysis. Further details on blood collection, storage, and measurement of 25(OH)D are available elsewhere [25].

*Offspring cord blood DNA methylation*

Details of the assessment of cord blood DNA methylation in both MoBa1 and MoBa2 have been described previously[22, 23]. Cord blood samples were collected at birth and frozen at -80 degrees C [24]. Bisulfite conversion was performed using the EZ-96 DNA Methylation kit (Zymo Research Corporation, Irvine, CA) and DNA methylation was measured at 485,577 CpGs in cord blood using Illumina’s Infinium HumanMethylation450 BeadChip^47^. Raw intensity (.idat) files were handled in R using the *minfi* package to calculate the methylation level at each CpG as the beta-value (β=intensity of the methylated allele (M)/(intensity of the unmethylated allele (U) + intensity of the methylated allele (M) + 100)) and the data were exported for quality control and processing. Probe and sample-specific quality control was performed in the MoBa1 and MoBa2 datasets separately. Control probes (N=65) and probes on X (N=11 230) and Y (N=416) chromosomes were excluded in the datasets. Remaining CpGs missing > 10% of methylation data were also removed (none in MoBa2). Samples indicated by Illumina to have failed or or have an average detection p-value across all probes < 0.05 (N=35 MoBa2) and samples with gender mismatch (N=8 MoBa2) were also removed. As for MoBa1 we accounted for the different probe designs by applying the intra-array normalization strategy Beta Mixture Quantile dilation (BMIQ)[26]. The Empirical Bayes method via *ComBat* was applied separately in each dataset for batch correction using the *sva* package in *R [27]*. Finally four samples determined to be ancestry outliers based on the principle components analysis of Illumina HumanCore genotype data were excluded from the analysis.

*Covariates*

Fetal sex, maternal smoking status, maternal age at birth, maternal pre-pregnancy BMI, maternal education, and parity were assessed via maternal questionnaire during pregnancy or from birth registry [28]. Maternal age was included as a continuous variable. Maternal smoking status during pregnancy was classified into three groups: non-smoker, stopped smoking in early pregnancy , and smoked throughout pregnancy. Maternal educational level was categorized into four groups based on years of education: less than high school/secondary school, high school/secondary school completion, some college or university, or 4 years of college/university or more. Estimation of six different white blood cell types (CD8+ T and CD4+ T lymphocytes, CD56+ natural killer cells, CD19+ B cells, CD14+ monocytes, and granulocytes) by Houseman method[15] was performed using the default implementation of the *estimateCellCounts* function in the minfi package[16].

**PREDO**

*Study Population*

The Prediction and Prevention of Preeclampsia and Intrauterine Growth Restriction (PREDO) study is prospective, multicenter longitudinal pregnancy cohort. The study recruited women with a singleton, intrauterine pregnancy who visited any of 10 study hospitals in Finland for an ultrasound screening at 12-13 weeks gestation between 2006 and 2010. Recruitment and characteristics of the cohort have been described elsewhere[29]. The sample is comprised of two subsamples, one of which recruited women with a known risk factor for preeclampsia and intrauterine growth restriction, and one of which recruited women regardless of risk factor status. To be eligible for the high-risk subsample, women must have had one of the following: preeclampsia in previous pregnancy, intrauterine growth restriction in previous pregnancy, gestational diabetes in previous pregnancy, pre-pregnancy obesity, chronic hypertension, Type 1 Diabetes, maternal age at birth < 20 years, maternal age at birth > 40 years, systemic lupus erythematosus, Sjogren’s syndrome, previous pregnancy with fetal demise at >22 weeks gestation or over 500g fetal weight. 110 women without any known risk factors were included to provide a normal pregnancy reference for blood samples. Exclusion criteria for the high-risk subsample included asthma diagnosed by a physician, allergy to ASA, tobacco smoking during pregnancy, previous peptic ulcer, previous placental ablation, inflammatory bowel disease, rheumatoid arthritis, haemophilia or thrombophilia, and multiple pregnancy. Of 5,332 recruited women, 4,785 were eligible and consented to participate. Of these, 1,083 were part of the high risk subsample, and 3,702 were from the general subsample. 4,777 of these pregnancies resulted in a live birth. For the current study, we restricted the sample to mother-child pairs with complete data on maternal mid-pregnancy Vitamin D, offspring DNA methylation, and all covariates, resulting in a total analytic sample of 301 mother-child dyads.

*Maternal mid-pregnancy Vitamin D*

Maternal 25(OH)D levels were measured from maternal serum samples taken at 14.43 to 22.86 weeks of gestation. 25(OH)D concentrations were measured with a fully automated IDS-iSYS analyzer (Immunodiagnostic Systems Ltd., Bolton, UK). The method was validated against liquid chromatography tandem mass spectrometry (LC-MS/MS) in house, as well as by the manufacturer. The two have good linear agreement, though themethod used by PREDO gives 0.72-fold lower results. Intra- and inter-assay CV% were <5% and 7%. The quality and accuracy of the serum 25(OH)D analysis in PREDO is validated on an ongoing basis by participation in the vitamin D External Quality Assessment Scheme (DEQAS, Charing Cross Hospital, London, UK). iSYS shows a 3% positive bias against all laboratory trimmed mean values and a 10% positive bias compared with NIST standards in international comparisons Within the sample, levels ranged between 26.80 and 139.9 nmol/L (mean: 69.58, SD: 19.04).

*Offspring DNA methylation*

Epigenome-wide methylation in cord blood samples was assessed using Illumina 450K microarrays. We randomized all samples on 96-well plates based on gender and maternal risk factors. Quality control was conducted using the R package *minfi*. Samples were excluded if they were duplicates, outliers in median intensities, or sex discrepant based on X and Y chromosomes. Probes on X or Y chromosomes, probes containing SNPs, cross-hybridizing probes, and CpG sites with low detection p-values in at least 50% of samples were also removed from the analysis. Maternal blood contamination was tested using methylation data at 10 CpGs independently identified as differentially methylated between cord and adult blood and indicative of maternal blood contamination. Samples with DNA methylation values above previously identified thresholds at >4 of the 10 sites were considered contaminated and removed from all future analyses. The final dataset contained data on 428,619 CpG sites. Betas were normalized using the *funnorm* function, incorporating the first 10 principal components from internal control probes. To check for batch effects, principal components were computed on these normalized betas. Two batches were significantly associated with main principal components and removed iteratively using the combat package. Additional details on these procedures are available elsewhere [30]. Cell type proportions were estimated using the Bakulski reference set [10].

*Covariates*

Gestational age at measurement of Vitamin D, fetal sex, season of vitamin D measurement, parity, and maternal age at delivery were assessed using data from the Finnish Medical Birth Register and Population Register. Maternal smoking was assessed using Finnish Medical Birth register data, and was categorized into 3 levels; no smoking during pregnancy, quit smoking in the first trimester, continued smoking through pregnancy. Maternal early-pregnancy BMI was based on weight and height measured at the first antenatal clinic visit (mean 8 weeks gestation) derived from the Finnish Medical Birth Register. Maternal education was assessed using self-report questionnaire at 12-13 weeks gestation, and was classified into primary education, secondary education, lower tertiary education, or upper tertiary education, as recommended by Statistics Finland. Ancestry was evaluated using offspring GWAS data, and 2 principal components with eigenvalues > 1 were included in the models. As PREDO is a highly ethnically homogenous sample of Finnish-speaking mothers from Southern Finland, this is likely sufficient to adjust for population stratification.

**Project Viva**

*Study Population*

Project Viva is a longitudinal pre-birth cohort established to examine the effects of events during early development on lifetime health outcomes[31]. Recruitment and characteristics of the cohort have been described in detail elsewhere [31]. Between April 1999 and November 2002, the study recruited women in early pregnancy from eight obstetric of Atrius Harvard Vanguard Medical associated, a multispecialty group practice in eastern Massachusetts. Exclusion criteria included multiple gestation, inability to answer questions in English, gestational age >= 22 weeks at recruitment, and plans to move away from the study area before delivery. Of 2670 enrolled participants, 2128 were still enrolled at delivery and had a live birth. For the current study, we restricted to mother-child pairs of white race/ethnicity with complete data on mid-pregnancy Vitamin D, offspring DNA methylation, and non-missing covariates resulting in a total analytic sample of 283 mother-child pairs.

*Maternal mid-pregnancy Vitamin D*

Vitamin D concentrations were assessed in plasma samples collected at 23.8-36.4 weeks gestation. Blood samples were initially refrigerated, then plasma was separated and stored at -80 degrees C. Samples were analyzed in duplicate for 25(OH)D concentration, using an automated chemiluminescence immunoassay[32] and a manual radioimmunoassay[33]. Values from the two assays were averaged to obtain more stable estimates of 25(OH)D level[34].

*Offspring Cord Blood Methylation*

Cord blood samples collected at birth were centrifuged within 24 hours of collection. Genomic DNA was extracted from nucleated cells using commercially available PureGene Kits (Fisher, Catalog Nos. A407-4, A416-4; Qiagen, Catalog Nos.158908, 158912, 158924), and frozen at -80 degrees C. Extracted DNA underwent bisulfite conversion using the Zymo EZ DNA Methylation kit (Zymo Research), and epigenome wide methylation was measured using the Illumina HM450K microarray. Data were preprocessed using the minfi package in R. Failed samples, replicates, non-CpG probes, and probes on X and Y chromosomes were removed. Data were checked for gender mismatch using X and Y chromosomes. CpG sites with low detection p-values were identified and flagged. Raw methylation values were Noob adjusted (background and dye bias adjusted), and methylation values were normalized using a beta-mixture quantile normalization method.

*Covariates*

Analyses in Project Viva were restricted to subjects of self-reported white race. Fetal sex, maternal smoking status, maternal age at enrollment, early pregnancy BMI, maternal education, and parity were assessed by self-administered questionnaires and interviews during pregnancy. Season of vitamin D measurement was categorized into 4 groups (Feb-Apr, May-Jul, Aug-Oct, Nov-Jan).

Supplementary Table 1: KEGG Pathways for genes annotated to top 7 CpGs most significantly associated with maternal vitamin D insufficiency (p value < 1 * 10^-5^)

| KEGG Term | Pathway | # genes on pathway | # differentially methylated genes | Overrepresentation p-value | False Discovery Rate |
| --- | --- | --- | --- | --- | --- |
| hsa00790 | Folate biosynthesis | 27 | 1 | 0.00862065662463912 | 1 |
| hsa00730 | Thiamine metabolism | 15 | 1 | 0.00992114168642132 | 1 |
| hsa04742 | Taste transduction | 85 | 1 | 0.0390575019893785 | 1 |
| hsa04924 | Renin secretion | 69 | 1 | 0.0401717628160003 | 1 |
| hsa05322 | Systemic lupus erythematosus | 120 | 1 | 0.0436262566909859 | 1 |
| hsa04260 | Cardiac muscle contraction | 83 | 1 | 0.0446749484402515 | 1 |
| hsa04930 | Type II diabetes mellitus | 46 | 1 | 0.0477135746023725 | 1 |
| hsa03082 | ATP dependent chromatin remodeling | 115 | 1 | 0.0511567600440762 | 1 |
| hsa01240 | Biosynthesis of cofactors | 152 | 1 | 0.051399190473087 | 1 |
| hsa04720 | Long term potentiation | 67 | 1 | 0.0515091099222682 | 1 |
| hsa050311 | Amphetamine addiction | 69 | 1 | 0.0532668210088278 | 1 |
| hsa04927 | Cortisol synthesis and secretion | 65 | 1 | 0.0568458447868595 | 1 |
| hsa04217 | Necroptosis | 146 | 1 | 0.0569354705268843 | 1 |
| hsa04929 | GnRH secretion | 64 | 1 | 0.0570476967257694 | 1 |
| hsa04914 | Progesterone mediated oocyte maturation | 98 | 1 | 0.0585217845363595 | 1 |
| hsa04912 | GnRH signaling pathway | 93 | 1 | 0.0666972232297055 | 1 |
| hsa04114 | Oocyte meiosis | 127 | 1 | 0.0681685493819225 | 1 |
| hsa04613 | Neutrophil extracellular trap formation | 175 | 1 | 0.0691813791704696 | 1 |
| hsa04726 | Serotonergic synapse | 113 | 1 | 0.0710772627287712 | 1 |
| hsa04911 | Insulin secretion | 86 | 1 | 0.0721624649227718 | 1 |

Supplementary Table 2: KEGG Pathways for genes annotated to top 500 CpGs most significantly associated with maternal vitamin D insufficiency (p value < 1 * 10^-3^)

| KEGG Term | Pathway | # genes on pathway | # differentially methylated genes | Overrepresentation p-value | False Discovery Rate |
| --- | --- | --- | --- | --- | --- |
| hsa00010 | Glycolysis / Gluconeogenesis | 66 | 1 | 0.680867 | 1 |
| hsa00020 | Citrate cycle (TCA cycle) | 30 | 1 | 0.401709 | 1 |
| hsa00030 | Pentose phosphate pathway | 30 | 0 | 1 | 1 |
| hsa00040 | Pentose and glucuronate interconversions | 35 | 0 | 1 | 1 |
| hsa00051 | Fructose and mannose metabolism | 34 | 2 | 0.170658 | 1 |
| hsa00052 | Galactose metabolism | 30 | 1 | 0.484769 | 1 |
| hsa00053 | Ascorbate and aldarate metabolism | 30 | 0 | 1 | 1 |
| hsa00061 | Fatty acid biosynthesis | 17 | 1 | 0.368708 | 1 |
| hsa00062 | Fatty acid elongation | 27 | 0 | 1 | 1 |
| hsa00071 | Fatty acid degradation | 43 | 0 | 1 | 1 |
| hsa00100 | Steroid biosynthesis | 20 | 0 | 1 | 1 |
| hsa00120 | Primary bile acid biosynthesis | 17 | 0 | 1 | 1 |
| hsa00130 | Ubiquinone and other terpenoid-quinone biosynthesis | 11 | 0 | 1 | 1 |
| hsa00140 | Steroid hormone biosynthesis | 61 | 0 | 1 | 1 |
| hsa00190 | Oxidative phosphorylation | 121 | 1 | 0.843944 | 1 |
| hsa00220 | Arginine biosynthesis | 22 | 1 | 0.33572 | 1 |
| hsa00230 | Purine metabolism | 125 | 1 | 0.947466 | 1 |
| hsa00232 | Caffeine metabolism | 6 | 0 | 1 | 1 |
| hsa00240 | Pyrimidine metabolism | 55 | 3 | 0.069768 | 1 |
| hsa00250 | Alanine, aspartate and glutamate metabolism | 37 | 1 | 0.553022 | 1 |
| hsa00260 | Glycine, serine and threonine metabolism | 39 | 2 | 0.098902 | 1 |
| hsa00270 | Cysteine and methionine metabolism | 51 | 2 | 0.258133 | 1 |
| hsa00280 | Valine, leucine and isoleucine degradation | 48 | 0 | 1 | 1 |
| hsa00290 | Valine, leucine and isoleucine biosynthesis | 4 | 0 | 1 | 1 |
| hsa00310 | Lysine degradation | 62 | 6 | 0.006157 | 1 |
| hsa00330 | Arginine and proline metabolism | 50 | 2 | 0.202811 | 1 |
| hsa00340 | Histidine metabolism | 22 | 0 | 1 | 1 |
| hsa00350 | Tyrosine metabolism | 36 | 2 | 0.121176 | 1 |
| hsa00360 | Phenylalanine metabolism | 16 | 2 | 0.035238 | 1 |
| hsa00380 | Tryptophan metabolism | 41 | 1 | 0.459848 | 1 |
| hsa00400 | Phenylalanine, tyrosine and tryptophan biosynthesis | 6 | 1 | 0.083719 | 1 |
| hsa00410 | beta-Alanine metabolism | 31 | 0 | 1 | 1 |
| hsa00430 | Taurine and hypotaurine metabolism | 16 | 0 | 1 | 1 |
| hsa00440 | Phosphonate and phosphinate metabolism | 6 | 0 | 1 | 1 |
| hsa00450 | Selenocompound metabolism | 17 | 0 | 1 | 1 |
| hsa00470 | D-Amino acid metabolism | 6 | 0 | 1 | 1 |
| hsa00480 | Glutathione metabolism | 58 | 0 | 1 | 1 |
| hsa00500 | Starch and sucrose metabolism | 34 | 1 | 0.44334 | 1 |
| hsa00510 | N-Glycan biosynthesis | 52 | 2 | 0.263288 | 1 |
| hsa00511 | Other glycan degradation | 18 | 1 | 0.357648 | 1 |
| hsa00512 | Mucin type O-glycan biosynthesis | 34 | 1 | 0.588327 | 1 |
| hsa00513 | Various types of N-glycan biosynthesis | 41 | 1 | 0.599222 | 1 |
| hsa00514 | Other types of O-glycan biosynthesis | 45 | 3 | 0.121413 | 1 |
| hsa00515 | Mannose type O-glycan biosynthesis | 22 | 1 | 0.390041 | 1 |
| hsa00520 | Amino sugar and nucleotide sugar metabolism | 49 | 3 | 0.070144 | 1 |
| hsa00524 | Neomycin, kanamycin and gentamicin biosynthesis | 5 | 1 | 0.171886 | 1 |
| hsa00531 | Glycosaminoglycan degradation | 19 | 0 | 1 | 1 |
| hsa00532 | Glycosaminoglycan biosynthesis - chondroitin sulfate / dermatan sulfate | 21 | 0 | 1 | 1 |
| hsa00533 | Glycosaminoglycan biosynthesis - keratan sulfate | 14 | 0 | 1 | 1 |
| hsa00534 | Glycosaminoglycan biosynthesis - heparan sulfate / heparin | 24 | 0 | 1 | 1 |
| hsa00561 | Glycerolipid metabolism | 63 | 3 | 0.164464 | 1 |
| hsa00562 | Inositol phosphate metabolism | 73 | 3 | 0.255477 | 1 |
| hsa00563 | Glycosylphosphatidylinositol (GPI)-anchor biosynthesis | 26 | 0 | 1 | 1 |
| hsa00564 | Glycerophospholipid metabolism | 97 | 4 | 0.177276 | 1 |
| hsa00565 | Ether lipid metabolism | 50 | 2 | 0.191274 | 1 |
| hsa00590 | Arachidonic acid metabolism | 61 | 0 | 1 | 1 |
| hsa00591 | Linoleic acid metabolism | 30 | 0 | 1 | 1 |
| hsa00592 | alpha-Linolenic acid metabolism | 26 | 0 | 1 | 1 |
| hsa00600 | Sphingolipid metabolism | 53 | 1 | 0.658071 | 1 |
| hsa00601 | Glycosphingolipid biosynthesis - lacto and neolacto series | 28 | 0 | 1 | 1 |
| hsa00603 | Glycosphingolipid biosynthesis - globo and isoglobo series | 15 | 0 | 1 | 1 |
| hsa00604 | Glycosphingolipid biosynthesis - ganglio series | 15 | 0 | 1 | 1 |
| hsa00620 | Pyruvate metabolism | 47 | 3 | 0.040515 | 1 |
| hsa00630 | Glyoxylate and dicarboxylate metabolism | 30 | 3 | 0.008948 | 1 |
| hsa00640 | Propanoate metabolism | 32 | 1 | 0.501952 | 1 |
| hsa00650 | Butanoate metabolism | 26 | 0 | 1 | 1 |
| hsa00670 | One carbon pool by folate | 19 | 1 | 0.299065 | 1 |
| hsa00730 | Thiamine metabolism | 15 | 1 | 0.311579 | 1 |
| hsa00740 | Riboflavin metabolism | 8 | 0 | 1 | 1 |
| hsa00750 | Vitamin B6 metabolism | 6 | 0 | 1 | 1 |
| hsa00760 | Nicotinate and nicotinamide metabolism | 36 | 1 | 0.481457 | 1 |
| hsa00770 | Pantothenate and CoA biosynthesis | 21 | 0 | 1 | 1 |
| hsa00780 | Biotin metabolism | 3 | 0 | 1 | 1 |
| hsa00785 | Lipoic acid metabolism | 19 | 0 | 1 | 1 |
| hsa00790 | Folate biosynthesis | 27 | 1 | 0.349253 | 1 |
| hsa00830 | Retinol metabolism | 67 | 0 | 1 | 1 |
| hsa00860 | Porphyrin metabolism | 46 | 0 | 1 | 1 |
| hsa00900 | Terpenoid backbone biosynthesis | 22 | 0 | 1 | 1 |
| hsa00910 | Nitrogen metabolism | 17 | 0 | 1 | 1 |
| hsa00920 | Sulfur metabolism | 10 | 0 | 1 | 1 |
| hsa00970 | Aminoacyl-tRNA biosynthesis | 43 | 0 | 1 | 1 |
| hsa00980 | Metabolism of xenobiotics by cytochrome P450 | 77 | 0 | 1 | 1 |
| hsa00982 | Drug metabolism - cytochrome P450 | 71 | 0 | 1 | 1 |
| hsa00983 | Drug metabolism - other enzymes | 81 | 2 | 0.258373 | 1 |
| hsa01040 | Biosynthesis of unsaturated fatty acids | 27 | 0 | 1 | 1 |
| hsa01100 | Metabolic pathways | 1514 | 39 | 0.034291 | 1 |
| hsa01200 | Carbon metabolism | 113 | 5 | 0.056116 | 1 |
| hsa01210 | 2-Oxocarboxylic acid metabolism | 32 | 2 | 0.096333 | 1 |
| hsa01212 | Fatty acid metabolism | 56 | 1 | 0.73959 | 1 |
| hsa01230 | Biosynthesis of amino acids | 71 | 3 | 0.137663 | 1 |
| hsa01232 | Nucleotide metabolism | 81 | 3 | 0.19857 | 1 |
| hsa01240 | Biosynthesis of cofactors | 152 | 4 | 0.230418 | 1 |
| hsa01250 | Biosynthesis of nucleotide sugars | 37 | 3 | 0.045194 | 1 |
| hsa01521 | EGFR tyrosine kinase inhibitor resistance | 78 | 1 | 0.925277 | 1 |
| hsa01522 | Endocrine resistance | 96 | 2 | 0.791331 | 1 |
| hsa01523 | Antifolate resistance | 30 | 1 | 0.434613 | 1 |
| hsa01524 | Platinum drug resistance | 74 | 1 | 0.785279 | 1 |
| hsa02010 | ABC transporters | 45 | 0 | 1 | 1 |
| hsa03008 | Ribosome biogenesis in eukaryotes | 75 | 0 | 1 | 1 |
| hsa03010 | Ribosome | 132 | 0 | 1 | 1 |
| hsa03013 | Nucleocytoplasmic transport | 107 | 0 | 1 | 1 |
| hsa03015 | mRNA surveillance pathway | 94 | 0 | 1 | 1 |
| hsa03018 | RNA degradation | 77 | 2 | 0.399381 | 1 |
| hsa03020 | RNA polymerase | 34 | 0 | 1 | 1 |
| hsa03022 | Basal transcription factors | 43 | 0 | 1 | 1 |
| hsa03030 | DNA replication | 36 | 1 | 0.484253 | 1 |
| hsa03040 | Spliceosome | 129 | 1 | 0.893439 | 1 |
| hsa03050 | Proteasome | 45 | 0 | 1 | 1 |
| hsa03060 | Protein export | 31 | 1 | 0.446816 | 1 |
| hsa03082 | ATP-dependent chromatin remodeling | 115 | 2 | 0.648714 | 1 |
| hsa03083 | Polycomb repressive complex | 82 | 1 | 0.893625 | 1 |
| hsa03250 | Viral life cycle - HIV-1 | 63 | 0 | 1 | 1 |
| hsa03260 | Virion - Human immunodeficiency virus | 5 | 0 | 1 | 1 |
| hsa03264 | Virion - Flavivirus | 4 | 0 | 1 | 1 |
| hsa03265 | Virion - Ebolavirus, Lyssavirus and Morbillivirus | 12 | 1 | 0.195586 | 1 |
| hsa03266 | Virion - Herpesvirus | 8 | 1 | 0.186409 | 1 |
| hsa03267 | Virion - Adenovirus | 4 | 0 | 1 | 1 |
| hsa03271 | Virion - Rotavirus | 2 | 0 | 1 | 1 |
| hsa03320 | PPAR signaling pathway | 75 | 2 | 0.404922 | 1 |
| hsa03410 | Base excision repair | 44 | 1 | 0.493296 | 1 |
| hsa03420 | Nucleotide excision repair | 61 | 1 | 0.640204 | 1 |
| hsa03430 | Mismatch repair | 23 | 1 | 0.341179 | 1 |
| hsa03440 | Homologous recombination | 40 | 0 | 1 | 1 |
| hsa03450 | Non-homologous end-joining | 13 | 0 | 1 | 1 |
| hsa03460 | Fanconi anemia pathway | 52 | 1 | 0.624109 | 1 |
| hsa04010 | MAPK signaling pathway | 298 | 4 | 0.965409 | 1 |
| hsa04012 | ErbB signaling pathway | 84 | 1 | 0.92725 | 1 |
| hsa04014 | Ras signaling pathway | 234 | 2 | 0.986915 | 1 |
| hsa04015 | Rap1 signaling pathway | 210 | 3 | 0.939374 | 1 |
| hsa04020 | Calcium signaling pathway | 250 | 3 | 0.971835 | 1 |
| hsa04022 | cGMP-PKG signaling pathway | 165 | 5 | 0.439669 | 1 |
| hsa04024 | cAMP signaling pathway | 225 | 4 | 0.821111 | 1 |
| hsa04060 | Cytokine-cytokine receptor interaction | 281 | 6 | 0.18839 | 1 |
| hsa04061 | Viral protein interaction with cytokine and cytokine receptor | 98 | 2 | 0.303547 | 1 |
| hsa04062 | Chemokine signaling pathway | 192 | 2 | 0.924844 | 1 |
| hsa04064 | NF-kappa B signaling pathway | 104 | 2 | 0.628746 | 1 |
| hsa04066 | HIF-1 signaling pathway | 108 | 3 | 0.517763 | 1 |
| hsa04068 | FoxO signaling pathway | 129 | 3 | 0.618082 | 1 |
| hsa04070 | Phosphatidylinositol signaling system | 97 | 5 | 0.112394 | 1 |
| hsa04071 | Sphingolipid signaling pathway | 119 | 2 | 0.814858 | 1 |
| hsa04072 | Phospholipase D signaling pathway | 147 | 5 | 0.447041 | 1 |
| hsa04080 | Neuroactive ligand-receptor interaction | 362 | 7 | 0.479857 | 1 |
| hsa04110 | Cell cycle | 158 | 3 | 0.632312 | 1 |
| hsa04114 | Oocyte meiosis | 127 | 1 | 0.935742 | 1 |
| hsa04115 | p53 signaling pathway | 75 | 1 | 0.851433 | 1 |
| hsa04120 | Ubiquitin mediated proteolysis | 141 | 2 | 0.827705 | 1 |
| hsa04122 | Sulfur relay system | 8 | 0 | 1 | 1 |
| hsa04130 | SNARE interactions in vesicular transport | 32 | 0 | 1 | 1 |
| hsa04136 | Autophagy - other | 31 | 3 | 0.029495 | 1 |
| hsa04137 | Mitophagy - animal | 101 | 0 | 1 | 1 |
| hsa04140 | Autophagy - animal | 164 | 5 | 0.331557 | 1 |
| hsa04141 | Protein processing in endoplasmic reticulum | 168 | 3 | 0.610956 | 1 |
| hsa04142 | Lysosome | 132 | 1 | 0.929833 | 1 |
| hsa04144 | Endocytosis | 246 | 5 | 0.776062 | 1 |
| hsa04145 | Phagosome | 147 | 2 | 0.861796 | 1 |
| hsa04146 | Peroxisome | 83 | 1 | 0.736252 | 1 |
| hsa04148 | Efferocytosis | 154 | 3 | 0.765695 | 1 |
| hsa04150 | mTOR signaling pathway | 155 | 4 | 0.583366 | 1 |
| hsa04151 | PI3K-Akt signaling pathway | 347 | 8 | 0.686597 | 1 |
| hsa04152 | AMPK signaling pathway | 120 | 5 | 0.260849 | 1 |
| hsa04210 | Apoptosis | 134 | 1 | 0.953758 | 1 |
| hsa04211 | Longevity regulating pathway | 89 | 4 | 0.251771 | 1 |
| hsa04213 | Longevity regulating pathway - multiple species | 61 | 3 | 0.260691 | 1 |
| hsa04215 | Apoptosis - multiple species | 31 | 0 | 1 | 1 |
| hsa04216 | Ferroptosis | 41 | 0 | 1 | 1 |
| hsa04217 | Necroptosis | 146 | 1 | 0.925254 | 1 |
| hsa04218 | Cellular senescence | 155 | 1 | 0.987049 | 1 |
| hsa04260 | Cardiac muscle contraction | 83 | 3 | 0.256868 | 1 |
| hsa04261 | Adrenergic signaling in cardiomyocytes | 152 | 3 | 0.779942 | 1 |
| hsa04270 | Vascular smooth muscle contraction | 134 | 4 | 0.421304 | 1 |
| hsa04310 | Wnt signaling pathway | 172 | 2 | 0.967718 | 1 |
| hsa04330 | Notch signaling pathway | 61 | 3 | 0.256799 | 1 |
| hsa04340 | Hedgehog signaling pathway | 55 | 2 | 0.509269 | 1 |
| hsa04350 | TGF-beta signaling pathway | 107 | 1 | 0.936523 | 1 |
| hsa04360 | Axon guidance | 181 | 4 | 0.869476 | 1 |
| hsa04370 | VEGF signaling pathway | 59 | 1 | 0.797633 | 1 |
| hsa04371 | Apelin signaling pathway | 137 | 3 | 0.679285 | 1 |
| hsa04380 | Osteoclast differentiation | 133 | 1 | 0.946485 | 1 |
| hsa04390 | Hippo signaling pathway | 156 | 3 | 0.876866 | 1 |
| hsa04392 | Hippo signaling pathway - multiple species | 28 | 0 | 1 | 1 |
| hsa04510 | Focal adhesion | 201 | 8 | 0.23976 | 1 |
| hsa04512 | ECM-receptor interaction | 89 | 6 | 0.040432 | 1 |
| hsa04514 | Cell adhesion molecules | 150 | 3 | 0.792516 | 1 |
| hsa04520 | Adherens junction | 93 | 1 | 0.934233 | 1 |
| hsa04530 | Tight junction | 168 | 4 | 0.599976 | 1 |
| hsa04540 | Gap junction | 87 | 0 | 1 | 1 |
| hsa04550 | Signaling pathways regulating pluripotency of stem cells | 141 | 3 | 0.78571 | 1 |
| hsa04610 | Complement and coagulation cascades | 86 | 0 | 1 | 1 |
| hsa04611 | Platelet activation | 124 | 3 | 0.598303 | 1 |
| hsa04612 | Antigen processing and presentation | 69 | 1 | 0.857948 | 1 |
| hsa04613 | Neutrophil extracellular trap formation | 175 | 4 | 0.352671 | 1 |
| hsa04614 | Renin-angiotensin system | 23 | 1 | 0.271254 | 1 |
| hsa04620 | Toll-like receptor signaling pathway | 99 | 1 | 0.829996 | 1 |
| hsa04621 | NOD-like receptor signaling pathway | 173 | 2 | 0.795087 | 1 |
| hsa04622 | RIG-I-like receptor signaling pathway | 61 | 0 | 1 | 1 |
| hsa04623 | Cytosolic DNA-sensing pathway | 73 | 1 | 0.654031 | 1 |
| hsa04625 | C-type lectin receptor signaling pathway | 103 | 3 | 0.42575 | 1 |
| hsa04630 | JAK-STAT signaling pathway | 152 | 2 | 0.777427 | 1 |
| hsa04640 | Hematopoietic cell lineage | 92 | 1 | 0.830874 | 1 |
| hsa04650 | Natural killer cell mediated cytotoxicity | 115 | 1 | 0.931751 | 1 |
| hsa04657 | IL-17 signaling pathway | 92 | 3 | 0.192834 | 1 |
| hsa04658 | Th1 and Th2 cell differentiation | 90 | 1 | 0.920285 | 1 |
| hsa04659 | Th17 cell differentiation | 106 | 1 | 0.935066 | 1 |
| hsa04660 | T cell receptor signaling pathway | 118 | 1 | 0.949044 | 1 |
| hsa04662 | B cell receptor signaling pathway | 82 | 1 | 0.843086 | 1 |
| hsa04664 | Fc epsilon RI signaling pathway | 67 | 1 | 0.80728 | 1 |
| hsa04666 | Fc gamma R-mediated phagocytosis | 96 | 2 | 0.720638 | 1 |
| hsa04668 | TNF signaling pathway | 117 | 2 | 0.734557 | 1 |
| hsa04670 | Leukocyte transendothelial migration | 112 | 2 | 0.738478 | 1 |
| hsa04672 | Intestinal immune network for IgA production | 45 | 2 | 0.271846 | 1 |
| hsa04710 | Circadian rhythm | 33 | 0 | 1 | 1 |
| hsa04713 | Circadian entrainment | 97 | 1 | 0.954678 | 1 |
| hsa04714 | Thermogenesis | 219 | 4 | 0.657941 | 1 |
| hsa04720 | Long-term potentiation | 67 | 1 | 0.846904 | 1 |
| hsa04721 | Synaptic vesicle cycle | 78 | 2 | 0.60344 | 1 |
| hsa04722 | Neurotrophin signaling pathway | 119 | 4 | 0.383995 | 1 |
| hsa04723 | Retrograde endocannabinoid signaling | 140 | 1 | 0.969828 | 1 |
| hsa04724 | Glutamatergic synapse | 115 | 2 | 0.872386 | 1 |
| hsa04725 | Cholinergic synapse | 113 | 3 | 0.678915 | 1 |
| hsa04726 | Serotonergic synapse | 113 | 3 | 0.501261 | 1 |
| hsa04727 | GABAergic synapse | 89 | 2 | 0.736237 | 1 |
| hsa04728 | Dopaminergic synapse | 131 | 3 | 0.71223 | 1 |
| hsa04730 | Long-term depression | 60 | 1 | 0.824173 | 1 |
| hsa04740 | Olfactory transduction | 394 | 2 | 0.825134 | 1 |
| hsa04742 | Taste transduction | 85 | 2 | 0.469149 | 1 |
| hsa04744 | Phototransduction | 28 | 0 | 1 | 1 |
| hsa04750 | Inflammatory mediator regulation of TRP channels | 98 | 2 | 0.769857 | 1 |
| hsa04810 | Regulation of actin cytoskeleton | 228 | 5 | 0.716732 | 1 |
| hsa04814 | Motor proteins | 192 | 6 | 0.293337 | 1 |
| hsa04820 | Cytoskeleton in muscle cells | 232 | 4 | 0.875147 | 1 |
| hsa04910 | Insulin signaling pathway | 137 | 7 | 0.059084 | 1 |
| hsa04911 | Insulin secretion | 86 | 1 | 0.923026 | 1 |
| hsa04912 | GnRH signaling pathway | 93 | 1 | 0.913897 | 1 |
| hsa04913 | Ovarian steroidogenesis | 51 | 0 | 1 | 1 |
| hsa04914 | Progesterone-mediated oocyte maturation | 98 | 2 | 0.662139 | 1 |
| hsa04915 | Estrogen signaling pathway | 137 | 3 | 0.685233 | 1 |
| hsa04916 | Melanogenesis | 101 | 0 | 1 | 1 |
| hsa04917 | Prolactin signaling pathway | 70 | 2 | 0.540537 | 1 |
| hsa04918 | Thyroid hormone synthesis | 75 | 0 | 1 | 1 |
| hsa04919 | Thyroid hormone signaling pathway | 121 | 2 | 0.860848 | 1 |
| hsa04920 | Adipocytokine signaling pathway | 70 | 2 | 0.503754 | 1 |
| hsa04921 | Oxytocin signaling pathway | 153 | 2 | 0.934961 | 1 |
| hsa04922 | Glucagon signaling pathway | 105 | 1 | 0.904714 | 1 |
| hsa04923 | Regulation of lipolysis in adipocytes | 57 | 2 | 0.455135 | 1 |
| hsa04924 | Renin secretion | 69 | 2 | 0.462603 | 1 |
| hsa04925 | Aldosterone synthesis and secretion | 98 | 1 | 0.950853 | 1 |
| hsa04926 | Relaxin signaling pathway | 129 | 1 | 0.966385 | 1 |
| hsa04927 | Cortisol synthesis and secretion | 65 | 1 | 0.862155 | 1 |
| hsa04928 | Parathyroid hormone synthesis, secretion and action | 115 | 2 | 0.874784 | 1 |
| hsa04929 | GnRH secretion | 64 | 4 | 0.132264 | 1 |
| hsa04930 | Type II diabetes mellitus | 46 | 4 | 0.07052 | 1 |
| hsa04931 | Insulin resistance | 108 | 2 | 0.759855 | 1 |
| hsa04932 | Non-alcoholic fatty liver disease | 150 | 4 | 0.318139 | 1 |
| hsa04933 | AGE-RAGE signaling pathway in diabetic complications | 100 | 2 | 0.724375 | 1 |
| hsa04934 | Cushing syndrome | 155 | 1 | 0.990254 | 1 |
| hsa04935 | Growth hormone synthesis, secretion and action | 121 | 3 | 0.66849 | 1 |
| hsa04936 | Alcoholic liver disease | 133 | 2 | 0.744143 | 1 |
| hsa04940 | Type I diabetes mellitus | 41 | 2 | 0.406679 | 1 |
| hsa04950 | Maturity onset diabetes of the young | 26 | 1 | 0.492049 | 1 |
| hsa04960 | Aldosterone-regulated sodium reabsorption | 37 | 2 | 0.293333 | 1 |
| hsa04961 | Endocrine and other factor-regulated calcium reabsorption | 53 | 2 | 0.431886 | 1 |
| hsa04962 | Vasopressin-regulated water reabsorption | 44 | 0 | 1 | 1 |
| hsa04964 | Proximal tubule bicarbonate reclamation | 23 | 1 | 0.338848 | 1 |
| hsa04966 | Collecting duct acid secretion | 27 | 1 | 0.421236 | 1 |
| hsa04970 | Salivary secretion | 92 | 0 | 1 | 1 |
| hsa04971 | Gastric acid secretion | 76 | 2 | 0.646445 | 1 |
| hsa04972 | Pancreatic secretion | 101 | 1 | 0.887952 | 1 |
| hsa04973 | Carbohydrate digestion and absorption | 45 | 2 | 0.260034 | 1 |
| hsa04974 | Protein digestion and absorption | 102 | 3 | 0.43718 | 1 |
| hsa04975 | Fat digestion and absorption | 43 | 0 | 1 | 1 |
| hsa04976 | Bile secretion | 89 | 1 | 0.819835 | 1 |
| hsa04977 | Vitamin digestion and absorption | 26 | 0 | 1 | 1 |
| hsa04978 | Mineral absorption | 59 | 1 | 0.653911 | 1 |
| hsa04979 | Cholesterol metabolism | 50 | 0 | 1 | 1 |
| hsa04980 | Cobalamin transport and metabolism | 18 | 1 | 0.233953 | 1 |
| hsa05010 | Alzheimer disease | 366 | 8 | 0.531542 | 1 |
| hsa05012 | Parkinson disease | 249 | 2 | 0.953642 | 1 |
| hsa05014 | Amyotrophic lateral sclerosis | 347 | 5 | 0.772 | 1 |
| hsa05016 | Huntington disease | 288 | 5 | 0.673011 | 1 |
| hsa05017 | Spinocerebellar ataxia | 140 | 4 | 0.460353 | 1 |
| hsa05020 | Prion disease | 255 | 4 | 0.718823 | 1 |
| hsa05022 | Pathways of neurodegeneration - multiple diseases | 458 | 7 | 0.879409 | 1 |
| hsa05030 | Cocaine addiction | 49 | 2 | 0.434016 | 1 |
| hsa05031 | Amphetamine addiction | 69 | 2 | 0.572045 | 1 |
| hsa05032 | Morphine addiction | 91 | 1 | 0.948909 | 1 |
| hsa05033 | Nicotine addiction | 41 | 0 | 1 | 1 |
| hsa05034 | Alcoholism | 174 | 4 | 0.462991 | 1 |
| hsa05100 | Bacterial invasion of epithelial cells | 77 | 2 | 0.651479 | 1 |
| hsa05110 | Vibrio cholerae infection | 50 | 1 | 0.697342 | 1 |
| hsa05120 | Epithelial cell signaling in Helicobacter pylori infection | 70 | 0 | 1 | 1 |
| hsa05130 | Pathogenic Escherichia coli infection | 194 | 3 | 0.820973 | 1 |
| hsa05131 | Shigellosis | 240 | 5 | 0.634725 | 1 |
| hsa05132 | Salmonella infection | 245 | 3 | 0.890837 | 1 |
| hsa05133 | Pertussis | 76 | 0 | 1 | 1 |
| hsa05134 | Legionellosis | 56 | 0 | 1 | 1 |
| hsa05135 | Yersinia infection | 134 | 2 | 0.848646 | 1 |
| hsa05140 | Leishmaniasis | 74 | 1 | 0.817337 | 1 |
| hsa05142 | Chagas disease | 101 | 2 | 0.643546 | 1 |
| hsa05143 | African trypanosomiasis | 36 | 0 | 1 | 1 |
| hsa05144 | Malaria | 49 | 3 | 0.063584 | 1 |
| hsa05145 | Toxoplasmosis | 109 | 3 | 0.550415 | 1 |
| hsa05146 | Amoebiasis | 100 | 4 | 0.195195 | 1 |
| hsa05150 | Staphylococcus aureus infection | 89 | 2 | 0.379831 | 1 |
| hsa05152 | Tuberculosis | 168 | 2 | 0.874103 | 1 |
| hsa05160 | Hepatitis C | 147 | 2 | 0.8408 | 1 |
| hsa05161 | Hepatitis B | 153 | 2 | 0.887074 | 1 |
| hsa05162 | Measles | 129 | 3 | 0.467968 | 1 |
| hsa05163 | Human cytomegalovirus infection | 216 | 4 | 0.824048 | 1 |
| hsa05164 | Influenza A | 157 | 3 | 0.623549 | 1 |
| hsa05165 | Human papillomavirus infection | 321 | 9 | 0.540238 | 1 |
| hsa05166 | Human T-cell leukemia virus 1 infection | 219 | 5 | 0.702613 | 1 |
| hsa05167 | Kaposi sarcoma-associated herpesvirus infection | 184 | 1 | 0.989281 | 1 |
| hsa05168 | Herpes simplex virus 1 infection | 491 | 6 | 0.830185 | 1 |
| hsa05169 | Epstein-Barr virus infection | 189 | 4 | 0.71764 | 1 |
| hsa05170 | Human immunodeficiency virus 1 infection | 201 | 1 | 0.992221 | 1 |
| hsa05171 | Coronavirus disease - COVID-19 | 220 | 2 | 0.846409 | 1 |
| hsa05200 | Pathways in cancer | 522 | 8 | 0.965609 | 1 |
| hsa05202 | Transcriptional misregulation in cancer | 187 | 4 | 0.736027 | 1 |
| hsa05203 | Viral carcinogenesis | 193 | 5 | 0.504742 | 1 |
| hsa05204 | Chemical carcinogenesis - DNA adducts | 69 | 0 | 1 | 1 |
| hsa05205 | Proteoglycans in cancer | 202 | 2 | 0.981864 | 1 |
| hsa05206 | MicroRNAs in cancer | 297 | 7 | 0.399079 | 1 |
| hsa05207 | Chemical carcinogenesis - receptor activation | 213 | 4 | 0.715781 | 1 |
| hsa05208 | Chemical carcinogenesis - reactive oxygen species | 209 | 3 | 0.747284 | 1 |
| hsa05210 | Colorectal cancer | 86 | 1 | 0.910861 | 1 |
| hsa05211 | Renal cell carcinoma | 68 | 1 | 0.845175 | 1 |
| hsa05212 | Pancreatic cancer | 76 | 1 | 0.871523 | 1 |
| hsa05213 | Endometrial cancer | 58 | 1 | 0.852498 | 1 |
| hsa05214 | Glioma | 75 | 1 | 0.894908 | 1 |
| hsa05215 | Prostate cancer | 97 | 1 | 0.943464 | 1 |
| hsa05216 | Thyroid cancer | 37 | 0 | 1 | 1 |
| hsa05217 | Basal cell carcinoma | 63 | 1 | 0.860619 | 1 |
| hsa05218 | Melanoma | 72 | 1 | 0.871746 | 1 |
| hsa05219 | Bladder cancer | 41 | 0 | 1 | 1 |
| hsa05220 | Chronic myeloid leukemia | 76 | 1 | 0.881875 | 1 |
| hsa05221 | Acute myeloid leukemia | 67 | 1 | 0.847397 | 1 |
| hsa05222 | Small cell lung cancer | 92 | 3 | 0.441453 | 1 |
| hsa05223 | Non-small cell lung cancer | 72 | 3 | 0.406194 | 1 |
| hsa05224 | Breast cancer | 147 | 2 | 0.938508 | 1 |
| hsa05225 | Hepatocellular carcinoma | 169 | 1 | 0.991618 | 1 |
| hsa05226 | Gastric cancer | 149 | 2 | 0.937 | 1 |
| hsa05230 | Central carbon metabolism in cancer | 70 | 2 | 0.611128 | 1 |
| hsa05231 | Choline metabolism in cancer | 97 | 3 | 0.511316 | 1 |
| hsa05235 | PD-L1 expression and PD-1 checkpoint pathway in cancer | 89 | 1 | 0.877285 | 1 |
| hsa05310 | Asthma | 28 | 1 | 0.530493 | 1 |
| hsa05320 | Autoimmune thyroid disease | 42 | 1 | 0.712365 | 1 |
| hsa05321 | Inflammatory bowel disease | 63 | 1 | 0.787336 | 1 |
| hsa05322 | Systemic lupus erythematosus | 120 | 2 | 0.568272 | 1 |
| hsa05323 | Rheumatoid arthritis | 90 | 2 | 0.555241 | 1 |
| hsa05330 | Allograft rejection | 35 | 1 | 0.6777 | 1 |
| hsa05332 | Graft-versus-host disease | 38 | 1 | 0.679615 | 1 |
| hsa05340 | Primary immunodeficiency | 36 | 0 | 1 | 1 |
| hsa05410 | Hypertrophic cardiomyopathy | 99 | 3 | 0.509998 | 1 |
| hsa05412 | Arrhythmogenic right ventricular cardiomyopathy | 86 | 2 | 0.75211 | 1 |
| hsa05414 | Dilated cardiomyopathy | 104 | 3 | 0.580714 | 1 |
| hsa05415 | Diabetic cardiomyopathy | 187 | 3 | 0.724274 | 1 |
| hsa05416 | Viral myocarditis | 66 | 2 | 0.648624 | 1 |
| hsa05417 | Lipid and atherosclerosis | 206 | 2 | 0.934144 | 1 |
| hsa05418 | Fluid shear stress and atherosclerosis | 140 | 2 | 0.809986 | 1 |

Supplementary Table 3: Previously identified associations with top 7 CpG sites most significantly associated with maternal vitamin D insufficiency

| CpG | Exposure | Outcome | Tissue | Study PMID |
| --- | --- | --- | --- | --- |
| cg00183100 | Age | DNA methylation | whole blood | 33450751 |
|  | Fetal age | DNA methylation | Fetal brain | 25650246 |
|  | Age | DNA methylation | Fetal liver, adult liver | 25282492 |
| cg01832218 | Age | DNA methylation | whole blood | 33450751 |
| cg03096811 | Mortality | DNA methylation | whole blood | 29419728 |
| cg13549966 | Age | DNA methylation | whole blood | 33450751 |
|  | Rheumatoid arthritis | DNA methylation | whole blood | 23334450 |
| cg13973942 | Maternal BMI | DNA methylation | cord blood | 29016858 |
|  | DNA methylation | nitrogen dioxide exposure | whole blood | 29410382 |
|  | Papuan ancestry | DNA methylation | whole blood | 32453742 |
| cg23641264 | DNA methylation | CRP | whole blood | 38692281 |
|  | Eosinophilia | DNA methylation | nasal polyp | 32795589 |
|  | Papuan ancestry | DNA methylation | whole blood | 32453742 |
|  | DNA methylation | BMI | whole blood | 34670603 |
|  | Waist circumference | DNA methylation | whole blood | 34670603 |
|  | Type 2 diabetes mellitus | DNA methylation | whole blood | 10.1101/2023.01.10.23284387 |
|  | Liver cirrhosis | DNA methylation | whole blood | 10.1101/2023.01.10.23284387 |
| cg24043604 | Fetal sex | DNA methylation | placenta | 34044884 |
| cg11644123 | Rheumatoid arthritis | DNA methylation | whole blood | 23334450 |
|  | Age | DNA methylation | whole blood | 33450751 |
| cg12825872 | Inflammatory bowel disease | DNA methylation | whole blood | 27886173 |
|  | Age | DNA methylation | whole blood | 33450751 |
|  | Ulcerative colitis | DNA methylation | whole blood | 27886173 |
|  | Crohn's disease | DNA methylation | whole blood | 27886173 |

Supplementary Table 3: Prevalence of maternal vitamin D deficiency (25(OH)D < 30 nmol/L) in included cohorts

| Cohort | Prevalence of vitamin D deficiency (25(OH)D < 30 nmol/L)  % (n) |
| --- | --- |
| Generation R | 8.1 (173) |
| ALSPAC | 8.4 (42) |
| MoBa 1 | Data not available from cohort |
| MoBa 2 | Data not available from cohort |
| PREDO | 0.0 (1) |
| Gen3G | 3.4 (6) |
| EAGeR | 0.5 (2) |
| Project Viva | 5.3 (15) |

Due to changes in data availability over time, estimates of the prevalence of vitamin D deficiency were not available for MoBa. However, previous studies in these cohorts suggest that the prevalence of maternal mid-pregnancy vitamin D insufficiency in MoBa was approximately 10.9% [35].

Works Cited

1. Fraser, A., et al., *Cohort profile: the Avon Longitudinal Study of Parents and Children: ALSPAC mothers cohort.* International journal of epidemiology, 2013. **42**(1): p. 97-110.

2. Boyd, A., et al., *Cohort profile: the ‘children of the 90s’—the index offspring of the Avon Longitudinal Study of Parents and Children.* International journal of epidemiology, 2013. **42**(1): p. 111-127.

3. Lawlor, D.A., et al., *Association of maternal vitamin D status during pregnancy with bone-mineral content in offspring: a prospective cohort study.* The Lancet, 2013. **381**(9884): p. 2176-2183.

4. Relton, C.L., et al., *Data resource profile: accessible resource for integrated epigenomic studies (ARIES).* International journal of epidemiology, 2015. **44**(4): p. 1181-1190.

5. Min, J.L., et al., *Meffil: efficient normalization and analysis of very large DNA methylation datasets.* Bioinformatics, 2018. **34**(23): p. 3983-3989.

6. Fortin, J.-P., et al., *Functional normalization of 450k methylation array data improves replication in large cancer studies.* Genome biology, 2014. **15**(11): p. 503.

7. Leek, J.T. and J.D. Storey, *Capturing heterogeneity in gene expression studies by surrogate variable analysis.* PLoS genetics, 2007. **3**(9).

8. Schisterman, E.F., et al., *A randomised trial to evaluate the effects of low‐dose aspirin in gestation and reproduction: design and baseline characteristics.* Paediatric and perinatal epidemiology, 2013. **27**(6): p. 598-609.

9. Mumford, S.L., et al., *Association of preconception serum 25-hydroxyvitamin D concentrations with livebirth and pregnancy loss: a prospective cohort study.* The Lancet Diabetes & Endocrinology, 2018. **6**(9): p. 725-732.

10. Bakulski, K.M., et al., *DNA methylation of cord blood cell types: applications for mixed cell birth studies.* Epigenetics, 2016. **11**(5): p. 354-362.

11. Guillemette, L., et al., *Genetics of Glucose regulation in Gestation and Growth (Gen3G): a prospective prebirth cohort of mother–child pairs in Sherbrooke, Canada.* BMJ open, 2016. **6**(2): p. e010031.

12. Switkowski, K.M., et al., *Cord blood vitamin D status is associated with cord blood insulin and c-peptide in two cohorts of mother-newborn pairs.* The Journal of Clinical Endocrinology & Metabolism, 2019. **104**(9): p. 3785-3794.

13. Houseman, E.A., et al., *DNA methylation arrays as surrogate measures of cell mixture distribution.* BMC Bioinformatics, 2012. **13**: p. 86.

14. Bakulski, K.M., et al., *DNA methylation of cord blood cell types: Applications for mixed cell birth studies.* Epigenetics, 2016. **11**(5): p. 354-62.

15. Jaffe, A.E. and R.A. Irizarry, *Accounting for cellular heterogeneity is critical in epigenome-wide association studies.* Genome Biol, 2014. **15**(2): p. R31.

16. Team, R.C., *A language and environment for statistical computing.* R Foundation for Statistical Computing, Vienna, Austria. ISBN 3-900051-07-0, URL <http://www.R-project.org/>. 2013.

17. Jaddoe, V.W.V., et al., *The Generation R Study: design and cohort profile.* European journal of epidemiology, 2006. **21**(6): p. 475.

18. Kooijman, M.N., et al., *The Generation R Study: design and cohort update 2017.* European Journal of Epidemiology, 2016. **31**(12): p. 1243-1264.

19. Vinkhuyzen, A.A.E., et al., *Prevalence and predictors of vitamin D deficiency based on maternal mid-gestation and neonatal cord bloods: The Generation R Study.* The Journal of steroid biochemistry and molecular biology, 2016. **164**: p. 161-167.

20. Miller, S.A. and D. Dd, *Polesky HF. A simple salting out procedure for extracting DNA from human nucleated cells.* Nucleic Acids Res, 1988. **16**(3): p. 1215.

21. Magnus, P., et al., *Cohort profile update: the Norwegian mother and child cohort study (MoBa).* International journal of epidemiology, 2016. **45**(2): p. 382-388.

22. Håberg, S.E., et al., *Maternal folate levels in pregnancy and asthma in children at age three years.* The Journal of allergy and clinical immunology, 2011. **127**(1): p. 262.

23. Joubert, B.R., et al., *DNA methylation in newborns and maternal smoking in pregnancy: genome-wide consortium meta-analysis.* The American Journal of Human Genetics, 2016. **98**(4): p. 680-696.

24. Rønningen, K.S., et al., *The biobank of the Norwegian Mother and Child Cohort Study: a resource for the next 100 years.* European journal of epidemiology, 2006. **21**(8): p. 619-625.

25. Magnus, M.C., et al., *Prospective study of maternal mid‐pregnancy 25‐hydroxyvitamin D level and early childhood respiratory disorders.* Paediatric and perinatal epidemiology, 2013. **27**(6): p. 532-541.

26. Teschendorff, A.E., et al., *A beta-mixture quantile normalization method for correcting probe design bias in Illumina Infinium 450 k DNA methylation data.* Bioinformatics, 2013. **29**(2): p. 189-196.

27. Leek, J.T., et al., *The sva package for removing batch effects and other unwanted variation in high-throughput experiments.* Bioinformatics, 2012. **28**(6): p. 882-883.

28. Magnus, P., et al., *Cohort profile: the Norwegian mother and child cohort study (MoBa).* International journal of epidemiology, 2006. **35**(5): p. 1146-1150.

29. Girchenko, P., et al., *Cohort Profile: Prediction and prevention of preeclampsia and intrauterine growth restriction (PREDO) study.* International journal of epidemiology, 2017. **46**(5): p. 1380-1381g.

30. Girchenko, P., et al., *Associations between maternal risk factors of adverse pregnancy and birth outcomes and the offspring epigenetic clock of gestational age at birth.* Clinical epigenetics, 2017. **9**(1): p. 49.

31. Oken, E., et al., *Cohort profile: project viva.* International journal of epidemiology, 2015. **44**(1): p. 37-48.

32. Ersfeld, D.L., et al., *Analytical and clinical validation of the 25 OH vitamin D assay for the LIAISON® automated analyzer.* Clinical biochemistry, 2004. **37**(10): p. 867-874.

33. Hollis, B.W., et al., *Determination of vitamin D status by radioimmunoassay with an 125I-labeled tracer.* Clinical chemistry, 1993. **39**(3): p. 529-533.

34. Burris, H.H., et al., *Vitamin D status and hypertensive disorders in pregnancy.* Annals of epidemiology, 2014. **24**(5): p. 399-403. e1.

35. Amberntsson, A., et al., *Vitamin D intake and determinants of vitamin D status during pregnancy in The Norwegian Mother, Father and Child Cohort Study.* Frontiers in Nutrition, 2023. **10**: p. 1111004.

36. Sullivan, S., et al., *Prenatal vitamin D status and risk of psychotic experiences at age 18 years—a longitudinal birth cohort.* Schizophrenia research, 2013. **148**(1-3): p. 87-92.
